# Thiazole peptidomimetics as chemical modulators of *KRAS* gene expression *via* G-quadruplex stabilization

**DOI:** 10.1039/d5cb00046g

**Published:** 2025-10-02

**Authors:** Debasmita Biswas, Ananta Gorai, Sandip Maiti, Ritapa Chaudhuri, Sayantan Pradhan, Jyotirmayee Dash

**Affiliations:** a School of Chemical Sciences, Indian Association for the Cultivation of Science Kolkata 700032 India ocjd@iacs.res.in https://iacs.res.in/athusers/index.php?navid=0&userid=IACS0034#219524

## Abstract

*KRAS* is one of the most frequently mutated oncogenes in human cancers and remains a challenging target for therapeutic intervention, often labeled “undruggable.” We herein synthesized triazole-containing peptidomimetics TTh1 and TTh2, to explore their selective interactions with DNA quadruplexes. Biophysical studies reveal that TTh2 with a prolinamide motif selectively binds to and stabilizes the *KRAS* G-quadruplex structure, resulting in marked suppression of the *KRAS* mRNA and protein levels in HeLa cells. This downregulation correlates with the inhibition of key downstream signaling pathways, including MAPK and Akt/mTOR, which are critical for cancer cell proliferation and survival. These results highlight the potential of G4-binding peptidomimetics as chemical tools for modulating oncogene expression through selective stabilization of promoter G-quadruplex structures.

## Abstract 2

Bis-thiazole peptidomimetic **TTh2** selectively binds and stabilizes the *KRAS* promoter G-quadruplex, leading to *KRAS* downregulation and inhibition of MAPK and Akt/mTOR pathways, demonstrating a novel strategy for targeting ''undruggable'' oncogenes.

## Graphical abstract

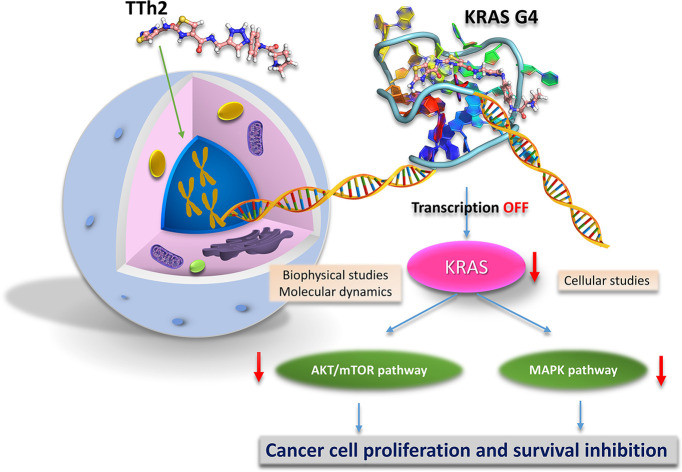

## Introduction

The *KRAS* proto-oncogene plays a central role in cellular communication networks that regulate growth, differentiation, and survival. As a member of the RAS family of proteins, *KRAS* encodes a GTPase that functions as a molecular switch, which transmits signals from cell surface receptors to intracellular pathways in response to external growth stimuli.^1^ In its unmutated, normal state, *KRAS* is essential for maintaining cellular homeostasis, regulating balanced cell proliferation and differentiation.^2^ However, mutations in the *KRAS* gene result in a constitutively active protein, leading to uncontrolled cell division—a hallmark of cancer.

The transformation of *KRAS* from a proto-oncogene to an active oncogene explains why *KRAS* mutations are implicated in various types of cancer, including pancreatic, colorectal, and lung cancers. Understanding the mechanism of *KRAS* action is crucial for the development of next-generation targeted therapies against *KRAS*-driven tumors. However, this remains a significant challenge due to the intratumoral heterogeneity and genetic mutability of *KRAS*.^3–5^ Once considered “undruggable” due to its structure and the nature of its mutations, *KRAS* has been resistant to targeting by most small-molecule inhibitors.^6^ The smooth surface of the protein and the absence of distinct binding pockets make it difficult for small molecules to effectively target it, in contrast to other oncogenes with more accessible binding sites.^7–9^ The diverse nature of *KRAS* mutations, such as G12C, G12D, G12V, and G13D, means that each mutation responds differently to drug treatment. As a result, a new therapeutic approach is needed to address the diverse nature of *KRAS* mutations and their varying responses to treatment. While progress has been made with the approval of sotorasib for treating cancers harboring the *KRAS* G12C mutation, other *KRAS* variants still lack targeted therapies.^10–17^ In this context, targeting *KRAS* DNA secondary structures, such as G-quadruplexes (G4s), offers an effective approach to cancer therapy compared to conventional strategies that focus on inhibiting the *KRAS* protein.

G-quadruplexes are four-stranded structures formed by G-rich nucleic acid sequences. They differ from the canonical B-form double helix and are held together by Hoogsteen hydrogen bonding, resulting in stacked G quartets. They are commonly found in the promoter regions of many transcriptional regulatory genes, including oncogenes and proto-oncogenes such as *KRAS*.^18–22^ The stabilization or disruption of such G4 conformations can be therefore utilized to modulate the transcription of the *KRAS* gene. G4-targeting drugs may offer greater selectivity, minimizing off-target effects and toxicity. This approach could also overcome resistance mechanisms often associated with protein inhibitors. With growing insights into the biological roles of G4s in cancer, including their involvement in gene regulation and genomic stability,^18^ targeting *KRAS* G4 structures using small molecules represents a promising frontier in cancer therapy.

Small molecules represent valuable therapeutic tools for probing and modulating G-quadruplex (G4)-associated cellular mechanisms.^23–30^ However, only a limited number of ligands have been reported to selectively bind and stabilize *KRAS* G4 structures.^27^ Herein, we have employed amide coupling as well as Cu(i)-catalyzed azide–alkyne cycloaddition to synthesize thiazole peptides TTh1 and TTh2. TTh2, featuring a prolinamide motif acts as a novel G4-binding ligand that selectively recognizes *KRAS* G4 structure and downregulates *KRAS* proto-oncogene expression.

## Results and discussion

### Ligand design and synthesis

Ligands such as multicarbazoles, porphyrin-based photosensitizers, isoquinoline alkaloids, and natural polyphenols have been reported to bind to the *KRAS* G-quadruplex (G4) based on biophysical and modeling studies.^31–34^ Moreover, compounds like berberine, coptisine, TMPyP4, and indolo[3,2-*b*]quinoline derivatives have been shown to reduce *KRAS* expression in cancer cells.^35–38^ However, most studies are limited to *in vitro* characterization, offering minimal insight into the downstream cellular effects or mechanisms. In this study, we have designed poly-thiazole ligands by incorporating a phenyl amino thiazole (TTh1) or a phenyl prolinamide (TTh2) moiety linked *via* a triazole unit (Scheme S3, SI). These ligands adopt a planar crescent-shaped geometry, which enables effective interactions with G-quadruplex DNA *via* π–π stacking and hydrogen bonding. Thiazole-containing peptides are present in numerous biologically active compounds and natural products.^28–30^ As amide bond surrogates, thiazoles contribute to peptide stability and enhance biological activity. We have previously reported thiazole-based ligands for targeting *c-MYC*^28,29^ and *c-KIT* G-quadruplexes.^30^ Other studies have also demonstrated interactions of thiazoles with various G4s *via* molecular docking and MD simulations.^39^ Compared to previously reported thiazole ligands, the bis-thiazole framework presented here exhibits high binding affinity and specificity towards the *KRAS* G4 structure, likely due to the extended conjugation and multivalent interaction abilities. Proline, known for influencing peptide conformation, was incorporated to enhance selective binding to a particular G-quadruplex.^40,41^ It likely positions the bis-thiazole scaffold for preferential recognition. The 1,2,3-triazole unit further stabilizes the interaction as a peptide bond isostere.

**Fig. 1:**
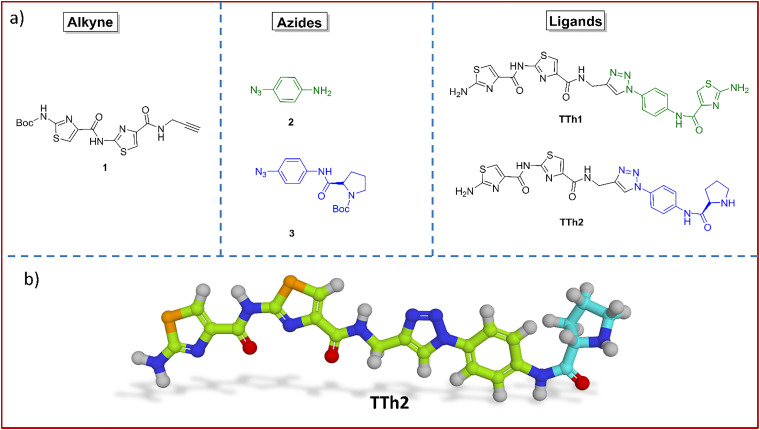
(a) Structure of alkyne 1, azides 2–3 and thiazole peptides (TTh1 and TTh2). (b) The energy optimized structure of TTh2. The rightmost cyan part is proline. For detailed synthetic procedures of TTh1 and TTh2, see the SI.

A bis-thiazole amide functionalized with an alkyne motif 1 was synthesized *via* consecutive amide coupling reactions followed by ester hydrolysis using thiazole amino acid building blocks (Scheme S1, SI). Azido prolinamide 3 was obtained *via* amide coupling of 4-azidoaniline 2 with N-Boc proline (Scheme S2, SI). The Cu(i)-catalyzed azide–alkyne cycloaddition (click reaction) between alkyne 1 and azide 2, followed by coupling with a thiazole amino acid, furnished the Boc-protected triazole-containing trithiazole peptidomimetic (Scheme S3, SI), which upon Boc deprotection with TFA provided TTh1. The Cu(i)-catalyzed click reaction of alkyne 1 with azide 3 containing a prolinamide motif and subsequent Boc deprotection, produced the triazole-containing bis-thiazole peptidomimetic TTh2 (Fig. 1a and Schemes S2 and S3, SI).

### Selective recognition of the *KRAS* G-quadruplex by TTh2

**Fig. 2:**
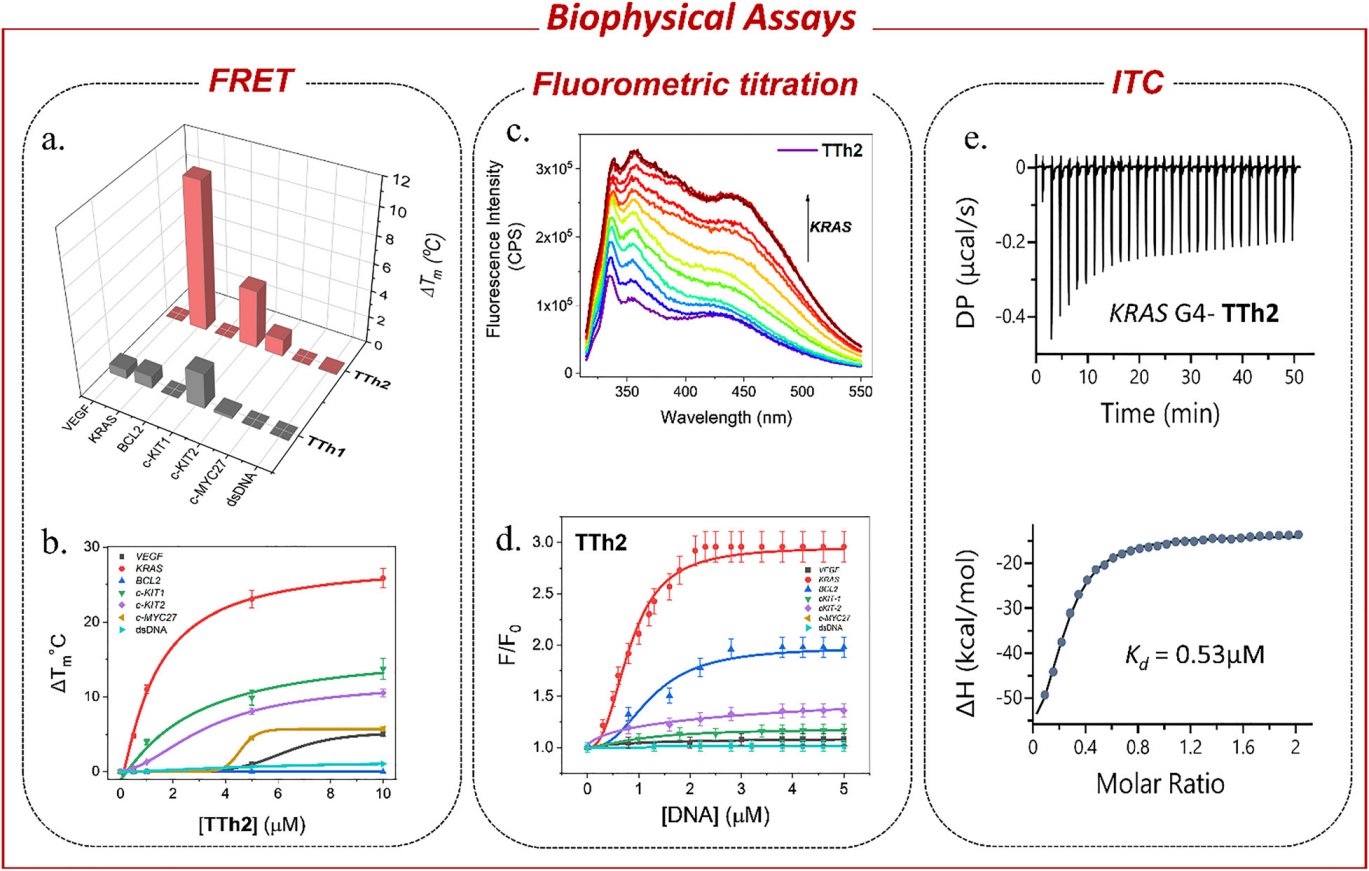
(a) 3D bar diagram showing the change in melting temperature (Δ*T*_m_) of DNA upon addition of 1 μM of TTh1 and TTh2. (b) FRET melting profiles of different G4s and ds-DNA (100 nM) upon interaction with 0–50 molar equivalents of TTh2 (0–10 μM) in 60 mM K^+^-cacodylate buffer, pH ∼ 7.4. (c) Fluorimetric titration of 2 μM of TTh2 with (∼0–10 equiv.) *KRAS* G4 DNA in 100 mM Tris-KCl buffer, pH ∼ 7.4. (d) Hill plot of TTh2 (2 μM) titrated with *VEGF*, *KRAS*, *BCL2*, *c-KIT1*, *c-KIT2*, *c-MYC27* G4 and ds DNA (∼0–10 equiv.). (e) Isothermal calorimetric titration of 5 μM *KRAS* G4 DNA with 50 μM of TTh2 in 100 mM Tris and 100 mM KCl buffer, pH ∼ 7.4.

A high-throughput FRET-based melting assay was performed using dual-labeled (5′ FAM–3′ TAMRA) G4 sequences including *VEGF*, *KRAS*, *BCL2*, *c-KIT1*, *c-KIT2*, *c-MYC27* and a control duplex DNA to assess the stabilization of the G4 structures by peptidomimetic ligands (Fig. 2a and b and Fig. S3–S4, Table S1, SI). This screening approach enabled the comparison of melting temperatures (*T*_m_) for DNA in the presence and absence of the ligand, providing a quantitative measure of ligand-induced stabilization. The results revealed that the proline-containing bis-thiazole conjugate TTh2 selectively stabilized *KRAS* (*T*_m_ ∼50 °C), *c-KIT1* (*T*_m_ ∼66 °C), and *c-KIT2* (*T*_m_ ∼70 °C) G4 structures in a dose-dependent manner. For *KRAS*, TTh2 exhibited Δ*T*_m_ values of 12.9 ± 1 °C and 24.7 ± 1 °C at 5 and 25 molar equivalents, respectively. In contrast, for *c-KIT1*, the Δ*T*_m_ values were ∼7 °C and ∼14 °C, while for *c-KIT2*, the corresponding values were only ∼1.2 °C and ∼7.6 °C at the same molar equivalents (Fig. 2a and b, Fig. S4, SI). TTh2 showed no effect on the melting temperatures of *BCL2* (*T*_m_ ∼74 °C), *VEGF* (*T*_m_ ∼76 °C), or *c-MYC27* (*T*_m_ ∼67 °C) G4s. For all studied G4s, negligible changes in melting temperature (Δ*T*_m_) were observed with TTh1, even at molar equivalents up to 50. Considerable stabilization of *KRAS* G4 (Δ*T*_m_ = 12.9 ± 1 °C) was observed at only 5 equivalents (1 μM) of TTh2. The melting temperature profiles of ds26 DNA (*T*_m_ ∼73 °C) remained unchanged upon interaction with any of these ligands. These findings suggest that TTh2 selectively stabilizes *KRAS* G4 compared to other G4 structures and ds26 DNA. Furthermore, the results highlight the importance of the prolinamide motif in TTh2 for its interaction with G4 structures within the synthesized thiazole peptides. To evaluate the selectivity of TTh2, FRET melting experiments were conducted using PhendC3, a well-known G4 stabilizer.^42–44^ PhendC3 increased the thermal stability (Δ*T*_m_ ∼10 °C) of all tested G4s, including *VEGF*, *KRAS*, *c-KIT1*, *c-KIT2*, and *BCL2*, even at a low concentration (1 μM, 10 equivalents), indicating a non-selective binding profile (Fig. S5, SI). In contrast, TTh2 selectively stabilized *KRAS* G4 with minimal effect on other G4 structures, demonstrating its preferential binding affinity. Next, a FRET competitive assay (Fig. S4b, SI) demonstrated the specific binding of peptide TTh2 to *KRAS* G4 over duplex DNA. No appreciable changes in the *T*_m_ values of G4 were observed upon the addition of a 50-fold molar excess of competitor duplex calf thymus (ct) DNA (10 μM) to a ligand concentration of 1 μM.

Fluorometric titration was then employed to determine the binding affinities (*K*_d_) of the ligands for various G4 structures (Fig. 2c and d, and Fig. S6, S7, SI). The titrations were carried out with an excitation wavelength of *λ*_ex_ = 300 nm. Both TTh1 and TTh2 exhibited two major emission peaks at approximately *λ*_max_ ≈ 354 nm and 427 nm. Incremental addition of *KRAS* G4 DNA resulted in approximately a 3-fold increase in the fluorescence intensity of TTh2 (*K*_d_ = 0.8 μM), whereas no substantial spectral change was observed during titration with dsDNA. When titrated with other G4 DNAs, TTh2 exhibited lower binding affinities compared to *KRAS* G4 DNA, with *K*_d_ values exceeding 1.5 μM. Similarly, the ligand TTh1 containing an aminothiazole sidechain showed a 7-fold increase in intensity (*K*_d_ = 1.8 μM) upon titration with *BCL2* DNA but exhibited a lower specificity and affinity for all G4 DNAs compared to TTh2. Despite its binding ability to *BCL2* G4 in the fluorescence titration assay, TTh1 did not stabilize *BCL2* G4, as shown by the FRET assay. Furthermore, TTh1 exhibited no significant binding potential with other G4s in either FRET or fluorometric titration assays. Consequently, TTh1 was excluded from further studies. Isothermal titration calorimetry (ITC) was performed to explore the thermodynamic properties of the binding interactions (Fig. 2e and Fig. S8, Table S2, SI). Peptide TTh2 exhibited a higher binding affinity for *KRAS* G4 DNA with a *K*_d_ value of ∼0.53 μM, Δ*G* = −8.56 kcal mol^−1^ and a 1 : 1 binding stoichiometry, while showing weaker interactions (limited hydrogen bonding) with other G4s (>5 μM). Consistent with the FRET and fluorometric results, TTh2 displayed comparatively lower binding affinities for *c-KIT1* and *c-KIT2*, with the *K*_d_ values of ∼3.3 μM and ∼2.6 μM, respectively. Both FRET and ITC studies confirmed that TTh2 exhibited negligible binding to duplex DNA. The binding isotherms generated from titrating TTh2 with G4 DNAs and control dsDNA (5 μM) indicated that all interactions were exothermic.

Circular dichroism (CD) spectroscopy was used to examine the structural response of *KRAS* G4 DNA (15 μM) upon titration with 0–10 equivalents of TTh2 under both salt-free and potassium-containing conditions (Fig. S9, SI). The spectra retained the characteristic features of a parallel G-quadruplex, including a positive peak near 262 nm and a negative peak around 240 nm. Although a modest gradual decrease in ellipticity at 262 nm was observed upon ligand addition, the overall spectral profile remained consistent. These results suggest that TTh2 binds to *KRAS* G4 without inducing significant changes to its global G-quadruplex topology.

### Molecular docking study reveals selectivity towards *KRAS* over other G4s

**Fig. 3:**
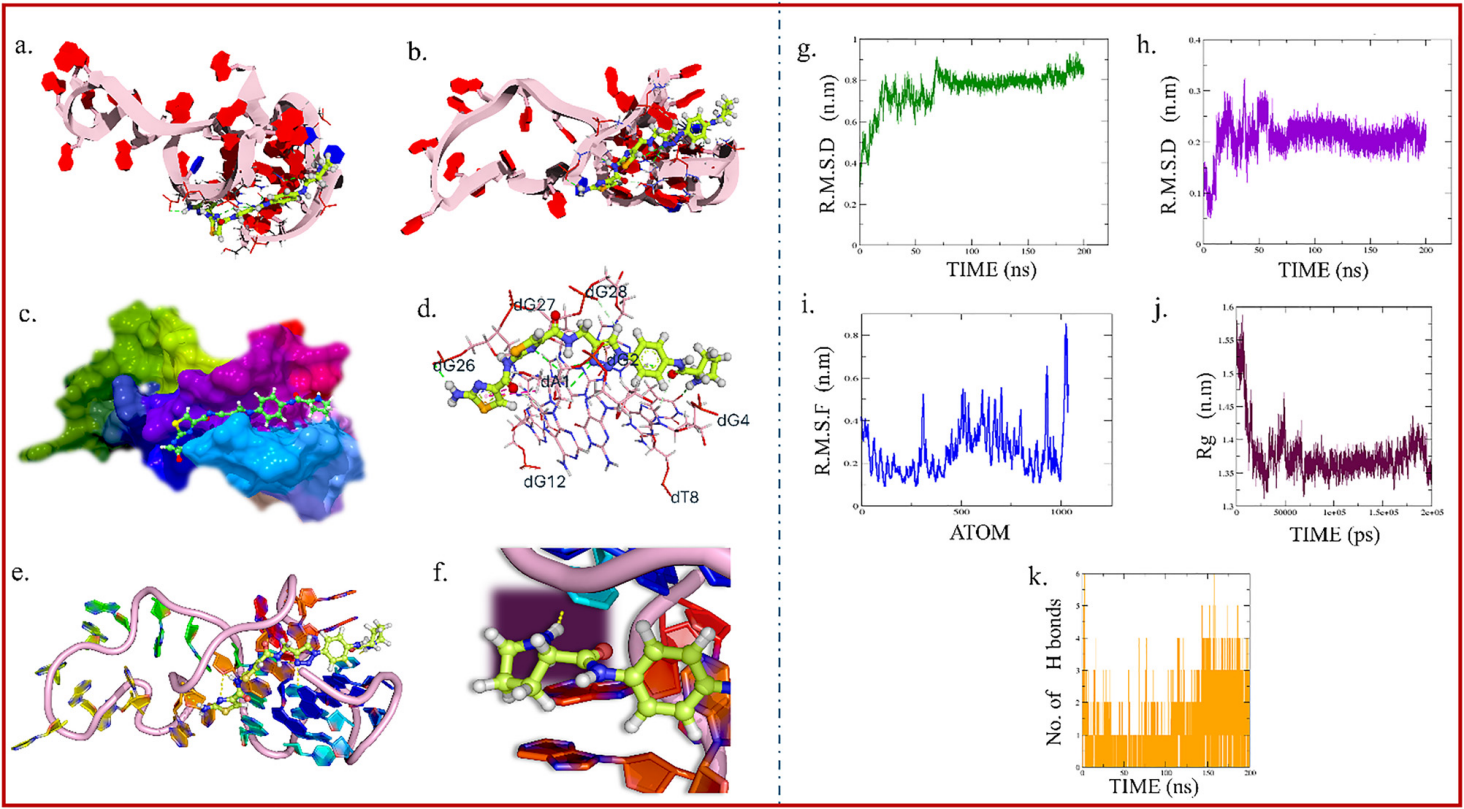
Visualization of the *KRAS* G-quadruplex and TTh2 interactions after docking (groove binding); (a) top view of the *KRAS* G-quadruplex DNA with TTh2; base pairs are represented as rings, and the DNA backbone is shown as a cartoon; (b) side view of the same structure, illustrating the spatial orientation of TTh2 relative to the G-quadruplex DNA; (c) surface model representation of the DNA, highlighting the regions accessible for interaction with TTh2; (d) detailed representation of interactions between TTh2 and DNA base pairs (dA, dT, dG, dC); (e) interaction between the proline motif of TTh2 and the *KRAS* G-quadruplex DNA; (f) the same interaction in different orientations. Interaction types, such as hydrogen bonds, are depicted as dotted lines, and the DNA base pairs surrounding TTh2 are labelled in blue with their two-letter codes. (g) The RMSD plot of DNA during simulation with TTh2; (h) the RMSD plot of ligand TTh2; (i) RMSF of DNA during simulation with TTh2; (j) the radius of gyration (*R*_g_) plot of DNA during simulation with TTh2; (k) the number of H-bonds between DNA complexed with TTh2. These results support stable groove binding of TTh2 and its role in maintaining the structural integrity of *KRAS* G4. Further details are provided in the SI.

The NMR structure of *KRAS* G-quadruplex DNA (PDB: 6T2G) was retrieved from the RCSB Protein Data Bank.^45^ The structure was prepared using MGL Tools by adding hydrogens, removing heteroatoms, and modeling missing loops.^46^ Docking with AutoDock Vina^47^ generated 20 binding poses, with TTh2 showing groove-binding behavior and a binding affinity of −9.5 kcal mol^−1^. Key interactions included eleven hydrogen bonds (1.85–3.22 Å) with bases such as dG4, dG28, dT8, and dA1, along with four π-interactions (Fig. 3a–f). The proline moiety of TTh2 contributed three essential hydrogen bonds, particularly with dG4, dG28, and dT8, stabilizing the complex within the DNA groove. The rigid cyclic structure of proline likely promotes groove binding by enhancing spatial complementarity and improving ligand stability. Additionally, docking studies with *c-MYC* and *BCL2* G4 structures resulted in less favourable docking scores, further supporting the binding preference of TTh2 for *KRAS* G4 (Fig. S10, SI).

To explore the binding affinity and stability of TTh2 with *KRAS* G-quadruplex DNA, a 200-ns molecular dynamics simulation was performed for groove binding modes. RMSD analysis showed early stabilization, with DNA and ligand RMSDs reaching a steady value at ∼0.80 nm and ∼0.25 nm, respectively. RMSF analysis revealed low flexibility at the G-quartet region and higher mobility at the loop and terminal regions. The radius of gyration (*R*_g_) remained steady at ∼1.4 nm, confirming the compactness of the G4 structure. Hydrogen bond analysis indicated stable interactions, with 3–5 H-bonds persisting after 130 ns, mainly involving dG4, dT8, dG28, and dA1 (Fig. 3g–k).

### TTh2 preferentially downregulates *KRAS* gene expression in HeLa cells

The cytotoxicity of TTh1 and TTh2 was evaluated against a cervical cancer cell line (HeLa) and a human embryonic (HEK) cell line using the MTT assay. In HeLa cells, ligands TTh1 and TTh2 demonstrated IC_50_ values of 28.2 ± 0.28 μM and 29.7 ± 0.35 μM, respectively (Fig. S11, SI). Furthermore, both the ligands displayed a significantly lower toxicity toward normal HEK cells with IC_50_ values exceeding 50 μM.

**Fig. 4:**
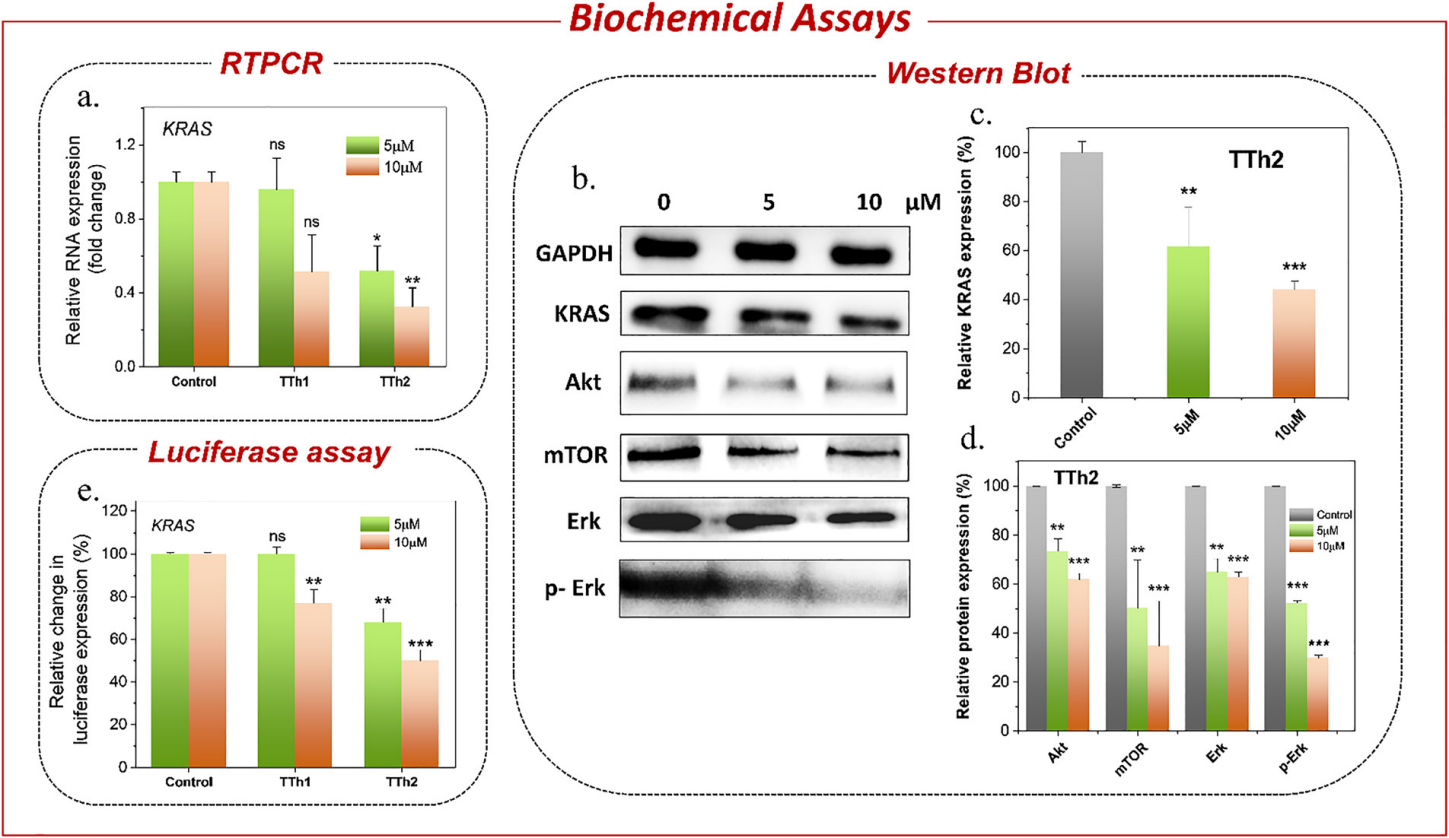
(a) qRT-PCR analysis of *KRAS* in HeLa cells after treatment with 5 and 10 μM TTh1 and TTh2 in DMSO for 24 h. (b) Western blot analysis of KRAS, Akt, mTOR, Erk, *p*-Erk and GAPDH proteins after treatment with TTh2 at 5 and 10 μM in HeLa cells. (c) Bar diagram representing the dose-dependent downregulation of the *KRAS* protein by 5 and 10 μM TTh2. (d) Bar diagram representing the dose-dependent expression of Akt, mTOR, Erk and *p*-Erk proteins after treatment with 5 and 10 μM of TTh2. (e) Relative luciferase activity (FF/RL) from the *KRAS* promoter-driven firefly luciferase plasmid normalized with the pRL-TK Renilla plasmid in HeLa cells treated with 5 μM and 10 μM TTh1 and TTh2.

Then, the *in vitro* effects of the ligands TTh1 and TTh2 on the expression of the *KRAS* gene, relative to housekeeping genes, were evaluated by qRT-PCR analysis in HeLa cells (Fig. 4a–d and Fig. S12, SI). After a 24 h treatment with TTh2, the expression levels of*KRAS* decreased to 0.5-fold (50% reduction) and 0.32-fold (68% reduction) at concentrations of 5 μM and 10 μM, respectively. Conversely, HeLa cells treated with 5 μM TTh1 showed no significant reduction in *KRAS* mRNA expression. However, at 10 μM, TTh1 lowered the expression levels to 0.51-fold, indicating a 49% reduction. The effect of TTh2 on *c-MYC* mRNA expression was also evaluated (Fig. S12b and c), and no significant changes were observed, indicating that TTh2 exhibits selectivity toward the *KRAS* gene.

The expression of *KRAS* was further silenced in a dose-dependent manner, as observed by the western blot analysis of the cell lysates collected 24 hours post-treatment with TTh2. TTh2 reduced the *KRAS* protein levels by 38.4% and 56% at concentrations of 5 μM and 10 μM, respectively (Fig. 4b–d and Fig. S13, SI). This reduction in the *KRAS* protein levels in HeLa cells led to the inhibition of key downstream signaling pathways including MAPK and Akt/mTOR, both critical for cancer cell proliferation and survival. The MAPK pathway is a major signaling pathway where *KRAS* is primarily activated by the interaction with RAF kinases and triggers a series of phosphorylation events that promote cellular growth and division. The reduction in *KRAS* expression resulted in decreased activation, as evidenced by a 70% reduction in ERK phosphorylation following treatment with 10 μM TTh2 (Fig. 4d). Similarly, TTh2 inhibited the Akt/mTOR pathway, a key regulator of cellular metabolism and growth, by significantly reducing the Akt/mTOR protein levels to 34.8% (at 10 μM [TTh2]) (Fig. 4d). This is particularly important as aberrant activation of the Akt/mTOR pathway is frequently associated with therapeutic resistance in many cancers. TTh2 may synergistically enhance the efficacy of existing treatments by targeting both the Akt/mTOR pathway and the MAPK pathway, overcoming the robust resistance mechanisms commonly observed in *KRAS*-mutant tumours.

**Fig. 5:**
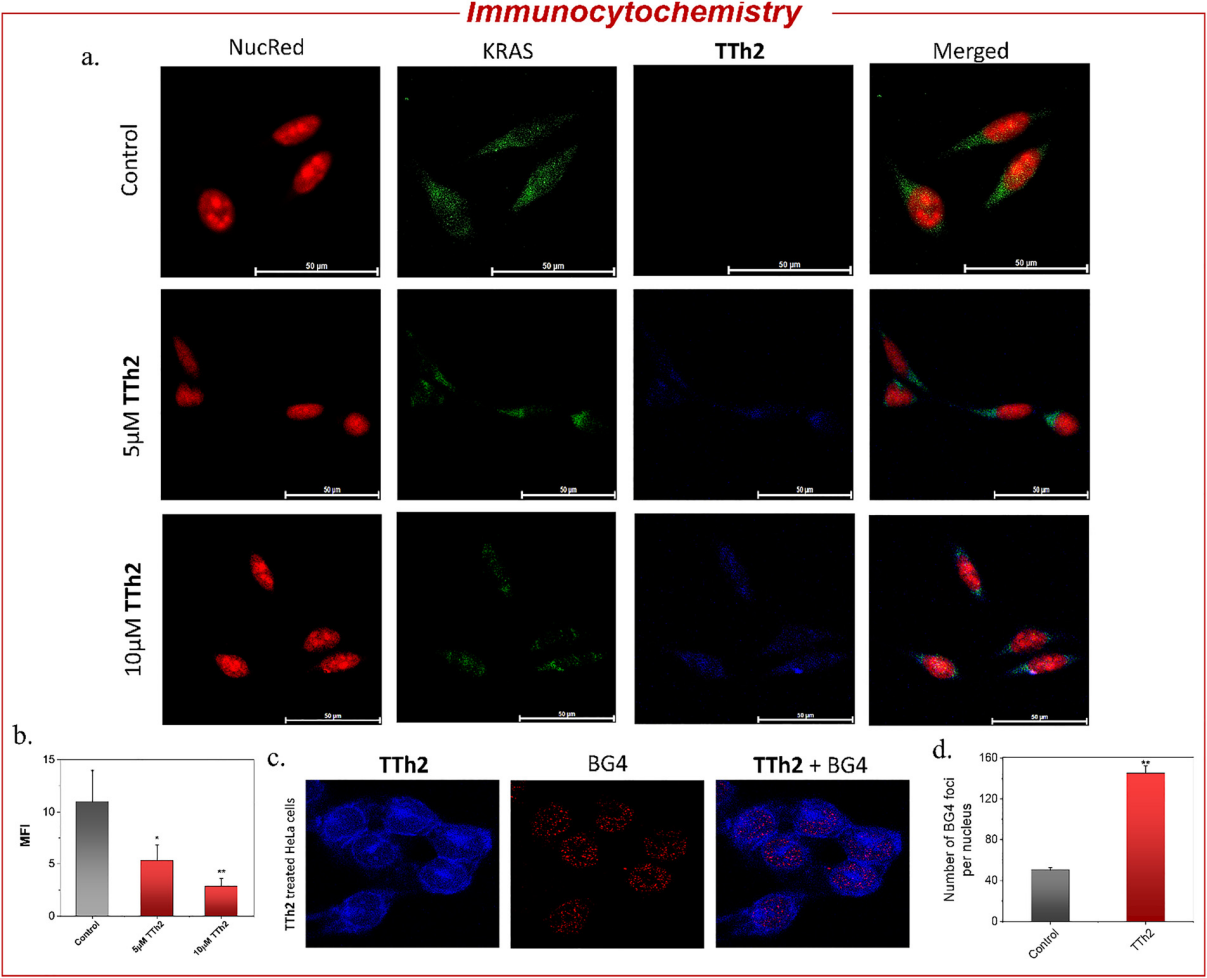
(a) Immunofluorescence analysis of *KRAS* protein expression in HeLa cells. The assay shows a dose-dependent decrease in green fluorescence, representing *KRAS* protein, within the cytoplasm with increasing TTh2 (blue signal) concentrations. Nuclei are counterstained with NucRed (red signal) as a reference for nuclear regions, imaged using a Leica DMI8 microscope. Scale bars: 50 μm. (b) bar diagram showing dose-dependent reduction in mean fluorescence intensity (MFI) in HeLa cells. (c) Immunostaining with TTh2 and BG4 antibodies: cells were stained with TTh2 (visualized under the blue filter), BG4 antibody (visualized under the red filter), and the merged image shows co-localization, imaged using a Leica DMI8 Stellaris 5 microscope. Scale bars: 10 μm. (d) Bar diagram showing an increase in BG4 foci on treatment with TTh2. Data are shown as mean ± SD; *P* < 0.05.

Next, to validate the results obtained from qRT-PCR and Western blot analysis, we performed an immunofluorescence assay using a *KRAS*-specific antibody in HeLa cells. The assay revealed a dose-dependent reduction in the green fluorescence intensity, representing *KRAS* protein, within the cytoplasm. This observation further confirms the downregulation of *KRAS* protein expression by 2-fold and 4-fold with increasing TTh2 concentrations (Fig. 5a and b, and Fig. S15, SI). The nuclei were counterstained with Propidium Iodide, indicated by the red signal, serving as a reference for the nuclear regions. These results further support the evidence of *KRAS* protein downregulation observed in previous experiments.

The dual luciferase assay further confirmed that the observed changes in *KRAS* gene expression were due to the stabilization of the *KRAS* promoter G4 by TTh2 (Fig. 4e). The PGL3-*KRAS* plasmid, containing the G4 promoter, was co-transfected with the Renilla luciferase plasmid pRL-TK, which contains a non-G4 promoter, into HeLa cells. These transfected HeLa cells were treated with 5 μM or 10 μM each of TTh1 and TTh2 for 36 hours. After normalizing the Renilla luciferase expression, the results showed that treatment with TTh2 reduced luciferase expression driven by the *KRAS* promoter by 50% compared to control cells (Fig. 4e). In contrast, treatment with TTh1 did not result in any significant change in *KRAS* luciferase expression. Furthermore, the effect of TTh2 was evaluated using a luciferase reporter plasmid driven by the *c-MYC* promoter. No significant change in luciferase activity (normalized to Renilla) was observed, further supporting the selective interaction of the thiazole ligand TTh2 with the *KRAS* promoter G-quadruplex (Fig. S14, SI). G4-specific staining in the immunofluorescence assays using BG4 antibodies revealed the presence of G4 structures in the cells through scattered punctate [red] nuclear staining. The nuclear localization of TTh2 in HeLa cells was visualized as a blue fluorescence. This observation was further supported by a 2.9-fold increase in BG4 foci in TTh2-treated HeLa cells compared to the untreated control (Fig. 5c and d, and Fig. S16, SI). The increase in foci strongly suggests that TTh2 binds to G4 structures within the cells.

## Conclusions

In summary, we have synthesized ligands TTh1 and TTh2 to explore the potential of peptidomimetics in targeting noncanonical DNA G-quadruplexes (G4). This work demonstrates that the peptidomimetic ligand TTh2, with bis-thiazole and prolinamide motifs, selectively stabilizes the *KRAS* G4 structure. High-throughput FRET-based melting assays revealed that TTh2 effectively stabilizes the *KRAS* G4 structure, leading to a notable increase in melting temperature (Δ*T*_m_) in a dose-dependent manner. This stabilization was further corroborated by competitive binding assays, fluorometric titration assays and isothermal titration calorimetry (ITC), which demonstrated a high binding affinity of TTh2 for *KRAS* G4, coupled with a high specificity for G-quadruplex DNA over duplex DNA. The therapeutic potential of TTh2 was further explored in HeLa cells, where selective downregulation of the *KRAS* levels was observed through qRT-PCR, western blot analysis and immunofluorescence assays. Treatment with TTh2 resulted in significant reductions in both *KRAS* mRNA and protein levels, which correlated with inhibition of crucial downstream signalling pathways, including MAPK and Akt/mTOR, essential for cell proliferation and survival. Moreover, dual-luciferase assays validated that the changes in *KRAS* expression were directly linked to the stabilization of the *KRAS* promoter G4 by TTh2. Immunofluorescence assays demonstrated an enhanced presence of G4s in HeLa cells, validating that TTh2 can interact with and stabilize G4s in a cellular context. In conclusion, the peptidomimetic ligand TTh2 represents a promising chemical probe for targeting G-quadruplex structures in gene regulatory regions, offering new opportunities to modulate *KRAS* expression and associated oncogenic pathways.

## Author contributions

J. D. conceptualized the project. J. D. and D. B. designed the experiments. A. G. and S. M. synthesized the compounds and performed HPLC, UV and CD spectroscopy. D. B. carried out FRET, fluorescence spectroscopy and ITC. D. B. performed the biological experiments. S. P. performed molecular dynamics and docking. R. C. performed microscopy using BG4 antibody. J. D. supervised the experiments. All authors analysed the data and J. D. and D. B. wrote the manuscript.

## Conflicts of interest

There are no conflicts to declare.

## Supplementary Material

CB-006-D5CB00046G-s001

## Data Availability

The data supporting this article have been included as part of the supplementary information (SI). Supplementary information: Synthesis of ligands, characterization of compounds and experimental methods. See DOI: https://doi.org/10.1039/d5cb00046g.
