# Supplementary material for: Thiazole peptidomimetics as chemical modulators of KRAS gene expression via G-quadruplex stabilization
Source: RSC Chem Biol. 2025 Oct 2;6(12):1885–92. doi: 10.1039/d5cb00046g (PMC12519992; doi:10.1039/d5cb00046g)
Supplement: CB-006-D5CB00046G-s001 [file CB-006-D5CB00046G-s001.pdf]

Supplementary Information (SI) for RSC Chemical Biology.
This journal is © The Royal Society of Chemistry 2025

Thiazole peptidomimetics as chemical modulators of *KRAS* gene expression via G-quadruplex stabilization

Debasmita Biswas [a], Ananta Gorai [a], Sandip Maiti[a], Ritapa Chaudhuri[a], Sayantan Pradhan[a], Jyotirmayee Dash*[a]

[a]School of Chemical Science, Indian Association for the Cultivation of Science, Jadavpur, Kolkata-700032, India

Email: ocjd@iacs.res.in

Table of contents

1.0	General information	S2
2.0	Synthesis of triazole peptidomimetics	S2
3.0	NMR spectra	S11
4.0	Mass analysis	S14
5.0	HPLC analysis	S15
6.0	UV/Vis spectroscopy	S16
7.0	FRET melting assay	S16
8.0	Fluorometric titration	S21
9.0	Isothermal calorimetry	S25
10.0	Circular dichroism spectroscopy	S27
11.0	Molecular dynamics simulation studies	S28
12.0	Cell cytotoxicity assay	S37
13.0	RNA extraction and RT-PCR experiments	S38
14.0	Western blot	S41
15.0	Transfection and dual luciferase assay	S43
16.0	Immunocytochemistry	S44
17.0	References	S46

1.0 General information

All experiments were carried out under an inert atmosphere of argon in flame-dried flasks. Solvents were dried using standard procedures. All starting materials were obtained from commercial suppliers and used as received. Products were purified by flash chromatography on silica gel (100-200 mesh, Merck). Unless otherwise stated, yields refer to analytical pure samples. NMR spectra were recorded in $CDCl_3$ unless otherwise stated. **^{1}H NMR** spectra were recorded at 500 MHz using Brüker ADVANCE 500 MHz and JEOL 400 MHz instruments at 298 K. Signals are quoted as δ values in ppm using residual protonated solvent signals as internal standard ($CDCl_3$: δ 7.26 ppm). Data is reported as follows: chemical shift, multiplicity (s = singlet, d = doublet, t = triplet, q = quartet, p = pentet, br = broad, m = multiplet), and coupling constants (Hz), integration. **^{13}C NMR** spectra were recorded on either a JEOL-400 MHz (100 MHz), or a Brüker ADVANCE 500 MHz (125 MHz) with complete proton decoupling. Chemical shifts (δ) are reported in ppm downfield from tetramethylsilane with the solvent as the internal reference ($CDCl_3$: δ 77.16 ppm). **HRMS** analyses were performed with Q-TOF YA263 high resolution (Water Corporation) instruments by +ve mode electrospray ionization.

2.0 Synthesis of triazole peptides

Thiourea **S1** was refluxed with bromo-pyruvic acid **S2** in dry ethanol to afford thiazole aminoester **S3**. Boc-protection of **S3**, followed by ester hydrolysis, provided N-Boc thiazole aminoacid **S5**. The thiazole aminoester **S6** was synthesized using amide coupling of **S3** and **S5** using HBTU and DIEA in 80% yield. Subsequent ester hydrolysis of **S6** gave the corresponding thiazole acid **S7**. Finally, **S7** was coupled with propargyl amine **S8** to afford the di-thiazole alkyne **1** in 85% yield (Scheme S1).

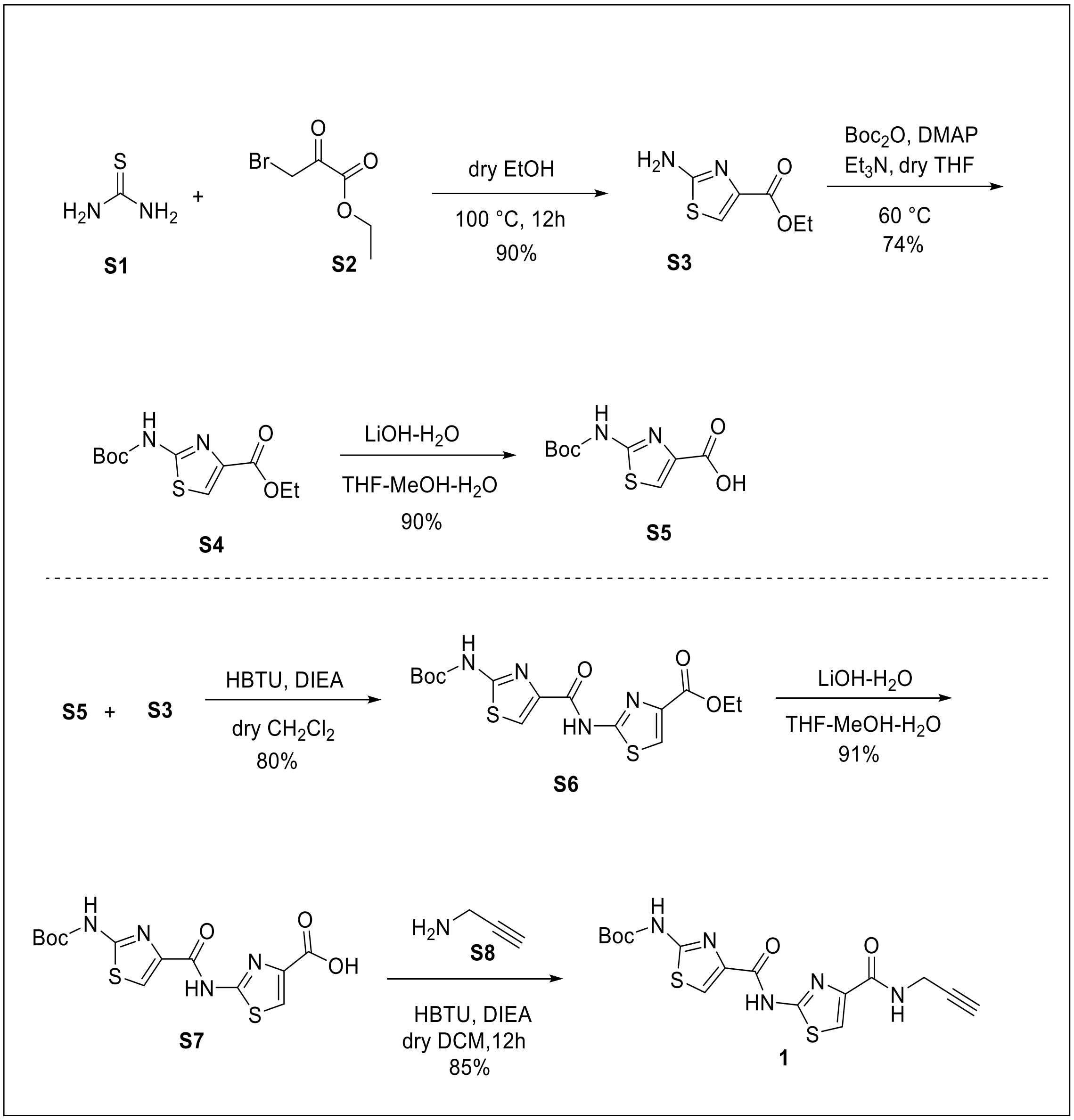

Scheme S1. Synthesis of di-thiazole alkyne **1.**

2.1 Synthesis of azide unit:

The amide coupling of N-Boc proline **S9** with 4-azidoaniline **2** was carried out using HBTU in the presence of DIEA in anhydrous CH_2Cl_2 to afford the azido prolinamide **3** (Scheme S2).

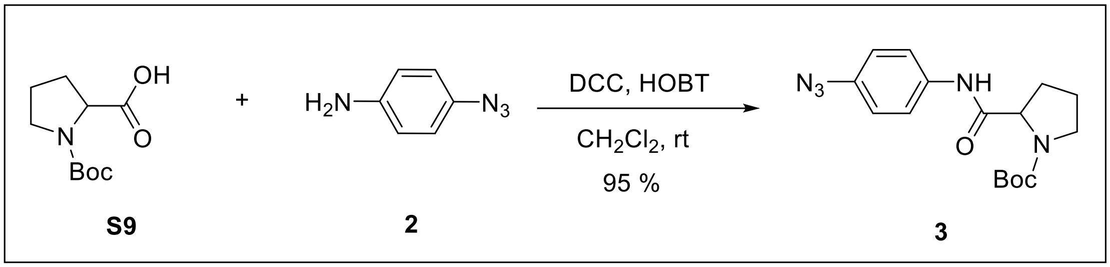

Scheme S2. Synthesis of azide units **3**.

2.2 Synthesis of triazolyl thiazole peptides (TTh1, TTh2) using click chemistry:

To synthesize **TTh1**, we first carried out click reaction between alkyne **1** and 4-azidoaniline **2** in presence of Cu(I) catalyst. The resulting product **S10** was coupled with thiazole amino acid **S5** using HBTU in the presence of DIEA in anhydrous dimethylformamide (DMF). The triazolyl thiazole containing peptide subsequently treated with TFA/CH_2Cl_2 for Boc deprotection to provide **TTh1** containing three thiazole motifs.

The Cu(I)-catalyzed cycloaddition of alkyne **1** with azide **3** followed by subsequent Boc-deprotection provided the triazole linked thiazole peptide **TTh2**, in high overall yields.

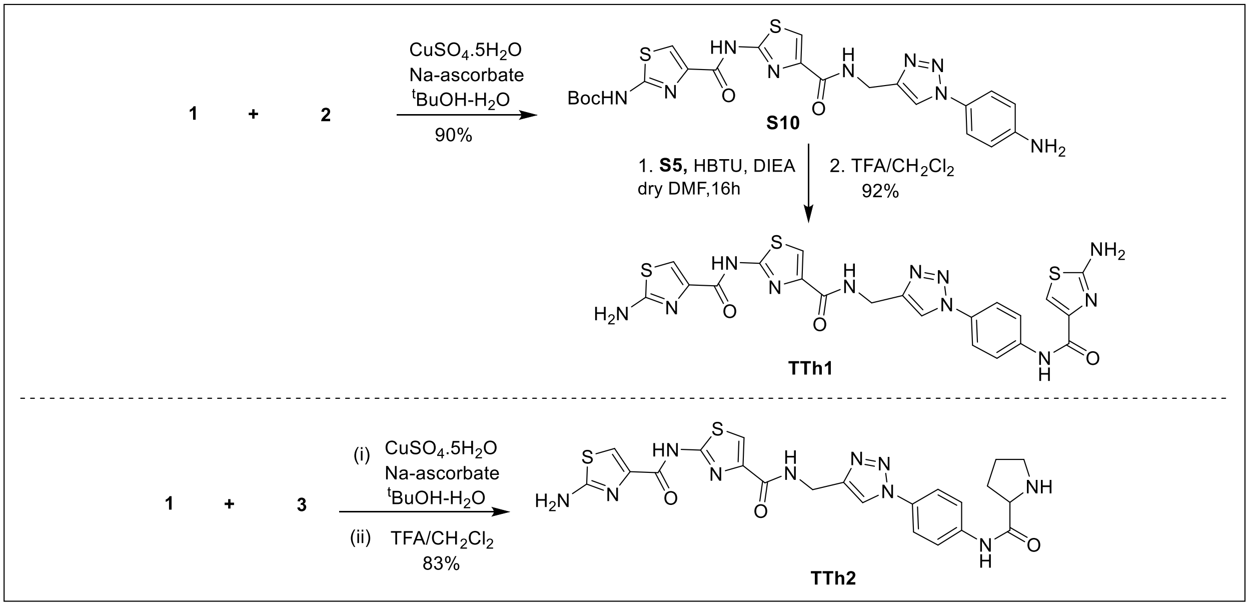

Scheme S3. Synthesis of thaizole derivatives (**TTh1** and **TTh2**) using Cu (I)-catalyzed azide-alkyne cycloaddition reaction.

Deprotection of the ethyl ester (P-1): To a solution of the respective ethyl ester protected thiazole derivative (1 eq) in THF/MeOH/H_2O = 3: 3: 1, LiOH-H_2O (3 eq) was added at 0 ºC. The reaction mixture was stirred for about 3-4 h at room temperature until starting material was fully consumed. Reaction was monitored by TLC. After completion of the reaction, solvent was dried in rotary evaporator. The crude obtained was dissolved in little amount of water followed by drop wise addition of saturated $KHSO_4$ solution to it under cold conditions that allowed the reaction mixture to get precipitated in acidic pH. The resulting solid precipitate was filtered and dried to afford the respective title hydrolyzed compound as solid in quantitative yield.

Procedure for the peptide coupling (P-2): All amide coupling reactions were carried out using *O*-(benzotriazol-1-yl)-*N*, *N*, *N′*, *N′*-tetramethyluronium hexafluorophosphate (HBTU) as a peptide coupling reagent, as this coupling reagent provided the desired coupled products in high yield. To a stirred solution of carboxylic acid component (1.1 eq) in dry CH_2Cl_2 or dimethylformamide (DMF), HBTU (1.5 eq) was added followed by the addition of N, *N′*-diisopropylethylamine (DIEA) (3 eq) at 0 ºC. After 10 minutes, the amine component (1 eq) was added at same temperature. The reactions were typically allowed to stir for 16-24 h at room temperature. After completion of the reaction, the reaction mixture was concentrated and the resulting residue was dissolved in ethylacetate and the organic layer was successively washed thrice with 1(N) HCl solution, saturated $NaHCO_3$ solution and brine. After drying with Na_2SO_4 and the solvents were removed under reduced pressure and the desired coupling products were purified by column chromatography.

Deprotection of the Boc-protecting group (P-3): The respective NH-Boc protected thiazole peptides were dissolved in CH_2Cl_2 and cooled to 0 °C. TFA (equal amount as the solvent) was added and the solution was allowed to warm to room temperature. The reaction mixture was stirred for about 3-4 h at room temperature until starting material was fully consumed. Reaction was monitored by TLC. After completion of the reaction, the solvent was removed under vacuum and the residue was washed with ether. The solid residue was dried under vacuum to provide the corresponding products.

Procedure for the Cu (I)-catalyzed azide-alkyne cycloaddition reaction (P-4): Thiazole alkyne **1** (1 equiv.) was dissolved in a 2:1 mixture of *t*-BuOH/H_2O (4 mL). Copper (II) sulphate

pentahydrate (0.1 equiv.) and sodium ascorbate (0.2 equiv.) were added and the solution was stirred for 10 min. The corresponding azide (l equiv.) was added and the mixture was allowed to stir at room temperature. After the completion of the reaction, mixture was concentrated. The crude product was purified by flash column chromatography (from CH_2Cl_2 (100%) to CH_2Cl_2/MeOH (10:1) to give the corresponding triazole derivatives **4** and **TTh2**.

Synthesis of ethyl 2-amino-4-thiazolecarboxylate (S3):[1] Ethyl bromopyruvate **S1** (5.31 g, 26.3 mmol) was added to a cold solution of thiourea **S2** (2 g, 26.3 mmol) in dry ethanol (5 mL) in a sealed tube. The resulting mixture was heated for 4 h at 100 °C. Upon cooling to room temperature, the reaction mixture was poured into ice water and brought to pH ~ 8 with aqueous sodium carbonate solution. The resulting solid precipitate was filtered, washed several times with water and air dried to obtain pure yellowish solid compound **S3** (4.07 g) in 90% yield. ^{1}H NMR (400 MHz, DMSO-d$_6$): 7.45 (s, 1H), 7.22 (s$_{br}$, 2H), 4.19 (q, *J* = 7.3 Hz, 2H), 1.25 (t, *J* = 6.7 Hz, 3H); ^{13}C NMR (100 MHz, DMSO-d$_6$): 168.2, 161.0, 142.2, 116.9, 60.1, 14.1; HRMS (ESI) calculated for $C_6H_9N_2O_2S$ $[M+H]^+$: 173.0379; found: 173.0391.

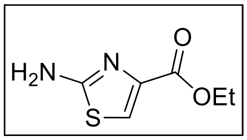

Synthesis of Boc-protected thiazole amino ester S4:[2] To a stirred solution of aminothiazole ester **S3** (2 g, 11.6 mmol) in tetrahydrofuran (40 mL) at 25 °C, triethylamine (2.12 mL, 15.2 mmol), 4-(dimethylamino) pyridine (140 mg, 1.16 mmol), and di-tert-butyl-dicarbonate (3 mL, 12.7 mmol) were sequentially added. The reaction mixture was heated to 60 °C. After 1 h, the reaction mixture was allowed to cool to 25 °C, and quenched with saturated aqueous ammonium chloride solution (50 mL). The two phases were separated, and the aqueous layer was extracted with ethyl acetate (3 × 20 mL). The combined organic layers were dried with Na_2SO_4 and concentrated under reduced pressure. The obtained residue was purified by column chromatography to afford pure thiazolyl carbamate **S4** (2.34 g, 74%) as a white solid. ^{1}H NMR (400 MHz, DMSO-d$_6$): 11.77 (s$_{br}$, 1H), 7.99 (s, 1H), 4.25 (q, *J* = 6.7 Hz, 2H), 1.47 (s, 9H), 1.27

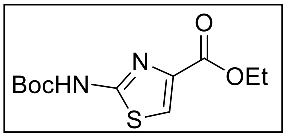

[1] Karuvalam, R. P.; Haridas, K. R.; Nayak, S. K.; Row, T. N. G.; Rajeesh, P.; Rishikesan, R.; Kumari, N. S. *Eur. J. Med. Chem.* **2012**, *49*, 172-182.

[2] Nicolaou, K. C.; Rhoades, D.; Wang, Y.; Bai, R.; Hamel, E.; Aujay, M.; Sandoval, J.; Gavrilyuk, J. *J. Am. Chem. Soc.* **2017**, *139*, 7318−7334.

(t, J = 7.3 Hz, 3H); ^{13}C NMR (100 MHz, DMSO-d$_6$): 160.9, 159.8, 153.0, 141.3, 122.2, 60.4, 27.8, 14.1; HRMS (ESI) calculated for $C_{11}H_{16}N_2O_4SNa$ [M+Na]$^+$: 295.0728; found: 295.0728.

Synthesis of Boc-protected thiazole amino acid (S5):[3] Using procedure **P-1**, Boc-protected thiazole ester **S4** (12.64 g, 45.3 mmol) was treated with LiOH·H_2O (5.7 g, 136 mmol) in a

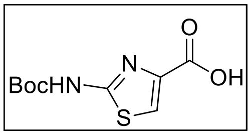

mixture of THF/MeOH/H_2O (60 mL) and stirred for 4 h. The reaction provided the corresponding thiazole acid **S5** (10.2 g, 90%) as a white solid. ^{1}H NMR (400 MHz, DMSO-d$_6$): 7.87 (s, 1H), 1.45 (s, 9H); ^{13}C NMR (100 MHz, DMSO-d$_6$): 162.7, 159.8, 153.3, 142.8, 121.9, 81.9, 28.1; HRMS (ESI) calculated for $C_9H_{12}N_2O_4SNa$ [M+Na]$^+$: 267.0415; found: 267.0403.

Synthesis of Boc-protected di-thiazole amide (S6):[3] Using procedure **P-2**, Boc-protected thiazole amino acid **S5** (6.24 g, 25.5 mmol), HBTU (13.19 g, 34.8 mmol), DIEA (12.12 mL, 69.6

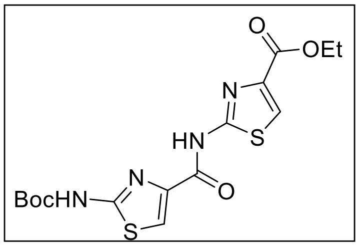

mmol), and thiazole amine **S3** (4 g, 23.2 mmol) were dissolved in dry CH_2Cl_2 (60 mL) and stirred for 16 h. The reaction afforded the desired product **S6** (7.4 g, 80%) as an off-white solid; ^{1}H NMR (500 MHz, CDCl$_3$): 10.68 (s$_{br}$, 1H), 9.01 (s$_{br}$, 1H), 7.85 (s, 1H), 7.82 (s, 1H), 4.27 (q, J = 7.3 Hz, 2H), 1.47 (s, 9H), 1.33 (t, J = 6.7 Hz, 3H); ^{13}C NMR (125 MHz, CDCl$_3$): 161.5, 160.2, 159.2, 157.8, 152.3, 142.5, 142.0, 122.5, 120.5, 83.3, 61.4, 28.2, 14.3; HRMS (ESI) calculated for $C_{15}H_{19}N_4O_5S_2$ [M+H]$^+$: 399.0791; found: 399.0778.

[3] Dutta, D.; Debnath, M.; Müller, D.; Paul, R.; Das, T.; Bessi, I.; Schwalbe, H.; Dash, J. *Nucleic Acid Res.* **2018***, 46,* 5355-5365.

Synthesis of Boc-protected di-thiazole acid (S7)[3]: Using procedure **P-1**, LiOH·H_2O (2.34 g, 55.7 mmol) and thiazole dipeptide **S6** (7.4 g, 18.6 mmol) in a THF/MeOH/H_2O mixture (40 mL) were stirred for 4 h, providing the corresponding thiazole acid **S7** (6.26 g, 91%) as a white solid.^{1}H NMR (500 MHz, DMSO-d$_6$): 12.29 (s_{br}, 1H), 11.82 (s_{br}, 1H), 8.20 (s, 1H), 8.03 (s, 1H), 1.49 (s, 9H); ^{13}C NMR (100 MHz, DMSO-d$_6$): 162.4, 160.1, 159.4, 157.7, 142.4, 142.3, 122.9, 120.5, 81.9, 27.9; HRMS (ESI) calculated for $C_{13}H_{14}N_4O_5S_2Na$ [M+Na]$^+$: 393.0303; found: 393.0289.

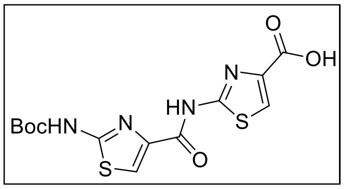

Synthesis of di-thiazole alkyne (1)[3]: To a stirred solution of acid **S7** (300 mg, 0.81 mmol) in dry CH_2Cl_2, HBTU (461 mg, 1.2 mmol) was added at once, followed by the addition of DIEA (0.4 mL, 2.4 mmol) at 0 ºC. After 10 minutes at 0 ºC, propargyl amine **S8** (0.062 mL, 0.97 mmol) was added. The reaction was allowed to stir for 24 h at room temperature. Upon completion of the reaction, the reaction mixture was concentrated, and the residue was dissolved in ethylacetate (30 mL). The organic layer was washed successively with saturated $NaHCO_3$ solution (3× 20 mL) and brine. The organic layer was dried with Na_2SO_4 and concentrated under reduced pressure. The resulting residue was purified by column chromatography to provide the desired compound **1** (281 mg, 85 %) as a white solid; ^{1}H NMR (400 MHz, DMSO-*d$_6$*): 11.91 (s_{br}, 1H), 11.77 (s_{br}, 1H), 8.51 (t, J = 5.4 Hz, 1H), 8.18 (s, 1H), 7.89 (s, 1H), 4.04 (d, J = 3.4 Hz, 2H), 3.12 (s, 1H), 1.51 (s, 9H); ^{13}C NMR (100 MHz, DMSO-d$_6$): 160.3, 160.1, 159.1, 157.4, 152.9, 144.2, 142.2, 120.5, 118.5, 81.9, 72.8, 28.1, 27.8; HRMS (ESI) calculated for $C_{16}H_{18}N_5O_4S_2$ [M+H$^+$]: 408.0795; found: 408.0787.

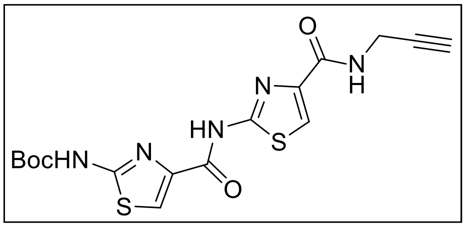

Synthesis of azido prolinamide (3)[4]: To an ice-cold suspension of *N*-Boc proline **S9** (1.0 g, 4.65 mmol) in dry CH_2Cl_2 (25 mL), DCC (1.06 g, 5.1 mmol) and HOBT (691 mg, 5.1 mmol) were added and the mixture was allowed to stir for 45 min. Then, 4-azidoaniline **2** (624 mg, 4.65 mmol, 1.0 equiv.) in 20 mL dry CH_2Cl_2 was added dropwise to the reaction mixture,

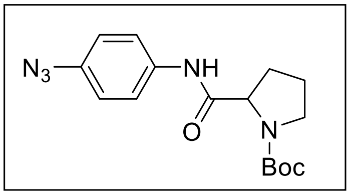

[4] Chakraborti, G., Paladhi, S., Mandal, T. and Dash, J. *J. Org. Chem.* **2018**. *83*(14), 7347-7359.

and stirred for 12 h. After complete consumption of the aniline azide **2** confirmed by TLC monitoring, the reaction mixture was filtered through Celite, washed with ethyl acetate (50 mL) and concentrated under vacuum. The product was purified by flash chromatography using hexane-ethylacetate (95:5 to 85:15) as eluent to afford the desired product **3** as a yellow solid (1.57 g, 95 %). ^{1}H NMR (400 MHz, $CDCl_3$): 9.61 (s_{br}, 1H), 7.48 (d, J = 9.4 Hz, 2H), 6.89 (s_{br}, 1H), 4.47 (s_{br}, 1H), 3.45-3.36 (m, 2H), 2.44 (s_{br}, 1H), 1.99-1.90 (m, 3H), 1.48 (s, 9H); ^{13}C NMR (100 MHz, $CDCl_3$): 170.0, 156.5, 135.5, 134.9, 120.8, 119.2, 80.9, 60.4, 47.3, 28.3, 27.5, 24.5; HRMS (ESI) calculated for [$C_{16}H_{22}N_5O_3K$] [M+K$^+$]: 370.1281; found: 370.1268.

Synthesis of di-thiazole triazole amine S10: Following P-4, a CuAAC reaction between the terminal alkynated thiazole peptide **1** (100 mg, 0.25 mmol), 4-azido aniline **2** (33 mg, 0.25 mmol) in presence of $CuSO_4.5H_2O$ (6.2 mg, 0.025 mmol), sodium ascorbate (10 mg, 0.050 mmol) in 4 mL ^{t}BuOH/H_2O (2:1) was carried out. The triazole product **S10** (122 mg, 90%) was obtained as a light brown solid. ^{1}H NMR (300 MHz, DMSO-d_6) δ 11.79 (s, 2H), 8.53 (t, J = 5.7 Hz, 1H), 8.37 (s, 1H), 8.19 (s, 1H), 7.90 (s, 1H), 7.46 (d, J = 8.8 Hz, 2H), 6.67 (d, J = 8.7 Hz, 2H), 5.47 (s, 2H), 4.59 (d, J = 5.7 Hz, 2H), 1.50 (s, 9H). 13C NMR (75 MHz, DMSO-d_6) δ 160.5, 160.2, 159.1, 157.4, 153.0, 149.3, 145.0, 144.5, 142.2, 126.0, 121.5, 120.7, 120.5, 118.3, 113.9, 81.9, 34.5, 27.9; HRMS (ESI) calculated for [$C_{22}H_{24}N_9O_4S_2$] [M+H$^+$]: 542.1393, found 542.1392.

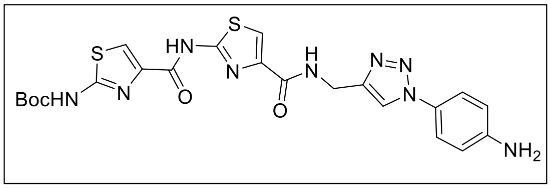

Synthesis of TTh1: Using procedure **P-2**, Boc-protected thiazole amino acid **S5** (60 mg, 0.2437 mmol), HBTU (126 mg, 0.33 mmol), DIEA (116 μL, 0.66 mmol), and triazole product **S10** (120 mg, 0.22 mmol) were dissolved in dry DMF (5 mL) and stirred for 16 h. The reaction afforded the desired Boc protected tri-thiazole triazole product as an off-white solid. After purification with flash column chromatography Boc deprotection using **P-3** yielded the final product **TTh1** (116 mg, 92%) as a light brown solid. ^{1}H NMR (500 MHz, DMSO-d_6) δ

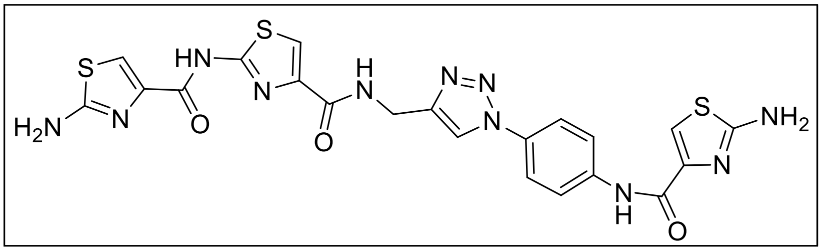

11.09 (s, 1H), 9.89 (s, 1H), 8.63 (s, 1H), 8.52 (s, 1H), 7.89 (d, J = 10.0 Hz, 2H), 7.80 (s, 1H), 7.77 (d, J = 10.0 Hz, 2H), 7.55 (s, 1H), 7.35 (s, 1H), 7.26 (s, 2H), 7.12 (s, 2H), 4.53 (s, 2H). ^{13}C NMR (126 MHz, DMSO-d_6) δ 168.6, 168.4, 162.4, 160.7, 159.6, 159.1, 145.8, 145.3, 144.5, 143.1, 138.8, 132.2, 121.0, 120.8, 120.5, 118.2, 115.2, 113.3, 34.5. HRMS (ESI) calculated for [$C_{21}H_{18}N_{11}O_3S_3$] [M+H$^+$]: 568.0756, found 568.0755.

Synthesis of TTh2: Following **P-4,** a CuAAC reaction between the terminal alkynated thiazole peptide **1** (100 mg, 0.25 mmol) and azido prolinamide **3** (83 mg, 0.25 mmol) in the presence of $CuSO_4.5H_2O$ (6.2 mg, 0.025 mmol), sodium ascorbate (10 mg, 0.050 mmol) in 4 mL

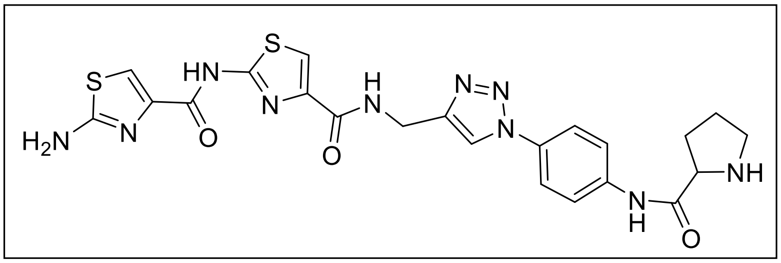

^{t}BuOH/H_2O (2:1) was carried out. After purification with flash column chromatography followed by Boc deprotection was performed using **P-3**. The triazole product **TTh2** (112 mg, 83%) was obtained as a yellow solid. ^{1}H NMR (400 MHz, DMSO-d_6) δ 10.79 (s, 1H), 9.35 (s, 1H), 8.69 (t, J = 5.8 Hz, 2H), 8.58 (s, 1H), 7.89 (d, J = 10.2 Hz, 2H), 7.77 (d, J = 8.9 Hz, 2H), 7.65 (s, 1H), 7.37 (s, 1H), 4.59 (d, J = 5.7 Hz, 2H), 4.35 (s, 1H), 3.37 (d, J = 7.1 Hz, 1H), 3.31 – 3.24 (m, 2H), 2.42 – 2.36 (m, 1H), 1.99 – 1.93 (m, 2H), 1.08 (t, J = 7.0 Hz, 2H). ^{13}C NMR (75 MHz, DMSO-d_6) δ 168.7, 167.2, 160.6, 158.7, 157.1, 145.9, 144.5, 142.4, 138.2, 132.6, 121.0, 120.8, 120.4, 118.3, 115.4, 59.8, 45.9, 34.5, 29.6, 23.6. HRMS (ESI) calculated for [$C_{22}H_{23}N_{10}O_3S_2$] [M+H$^+$]: 539.1396, found 539.1397.

3.0 NMR spectra

^{1}H and ^{13}C NMR of compound S10:

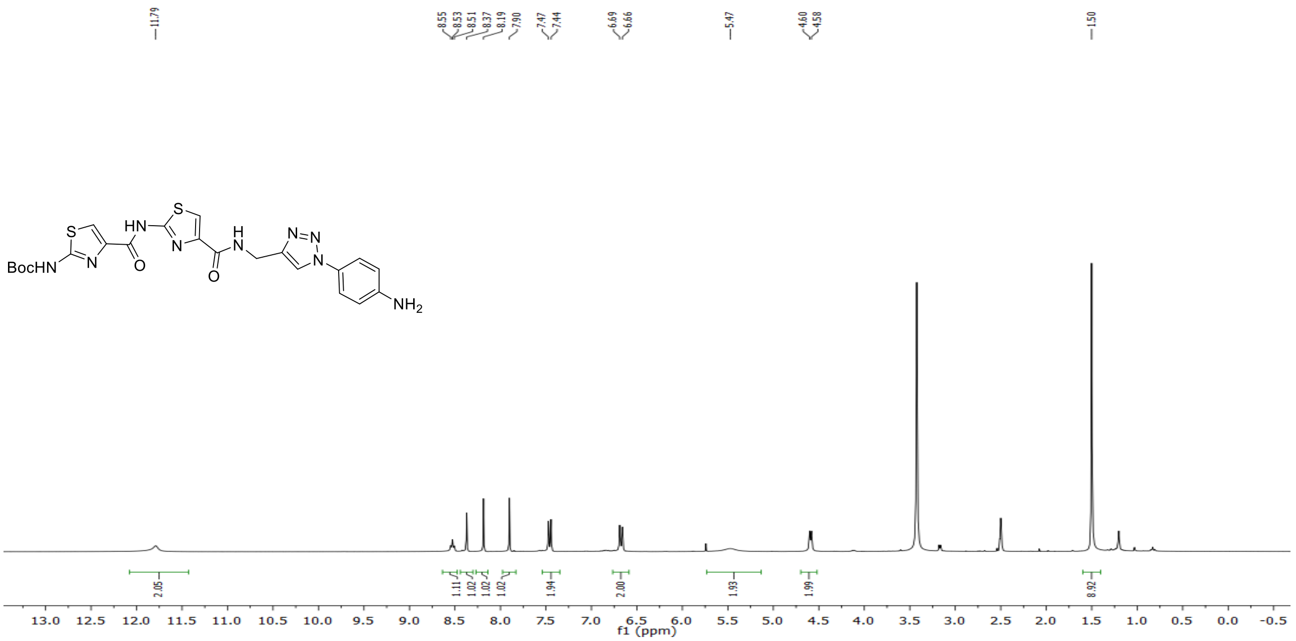

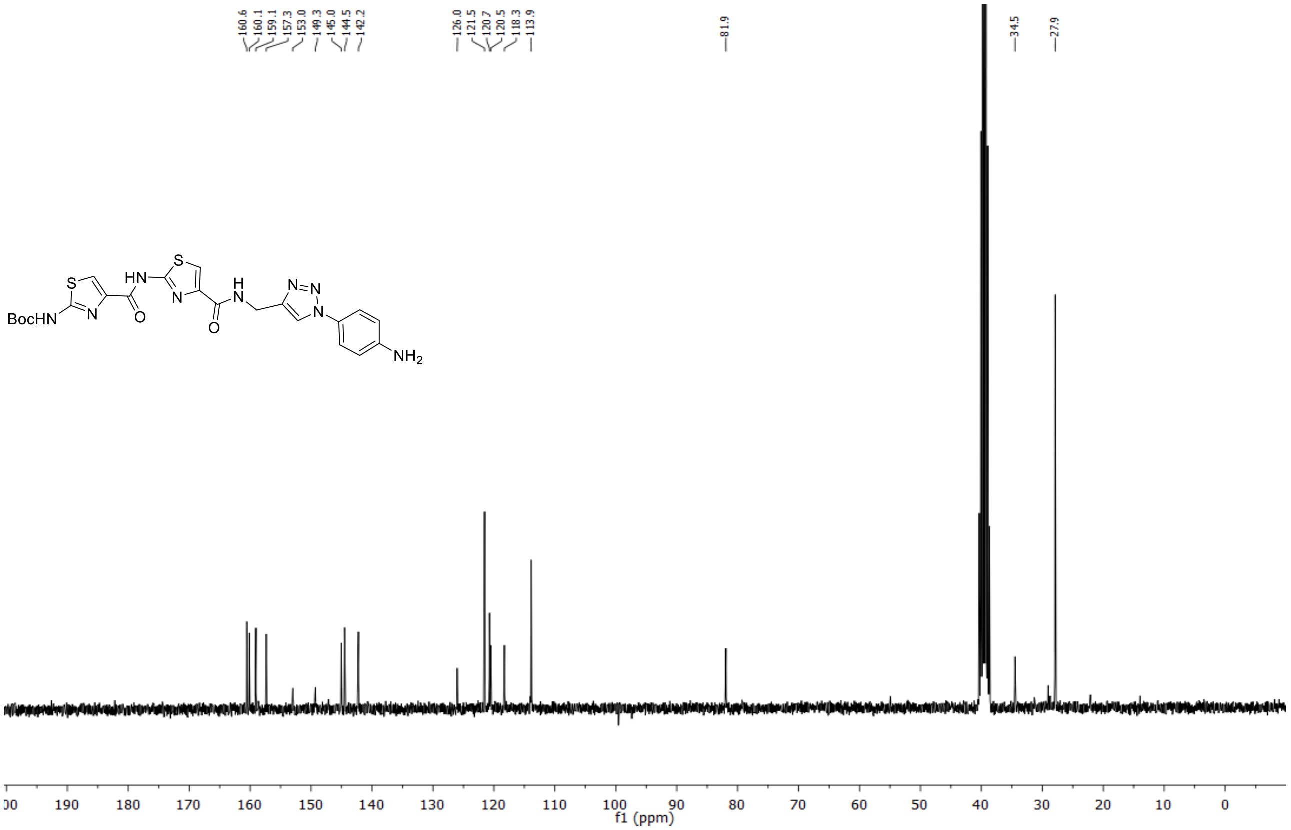

^{1}H and ^{13}C NMR of compound TTh1:

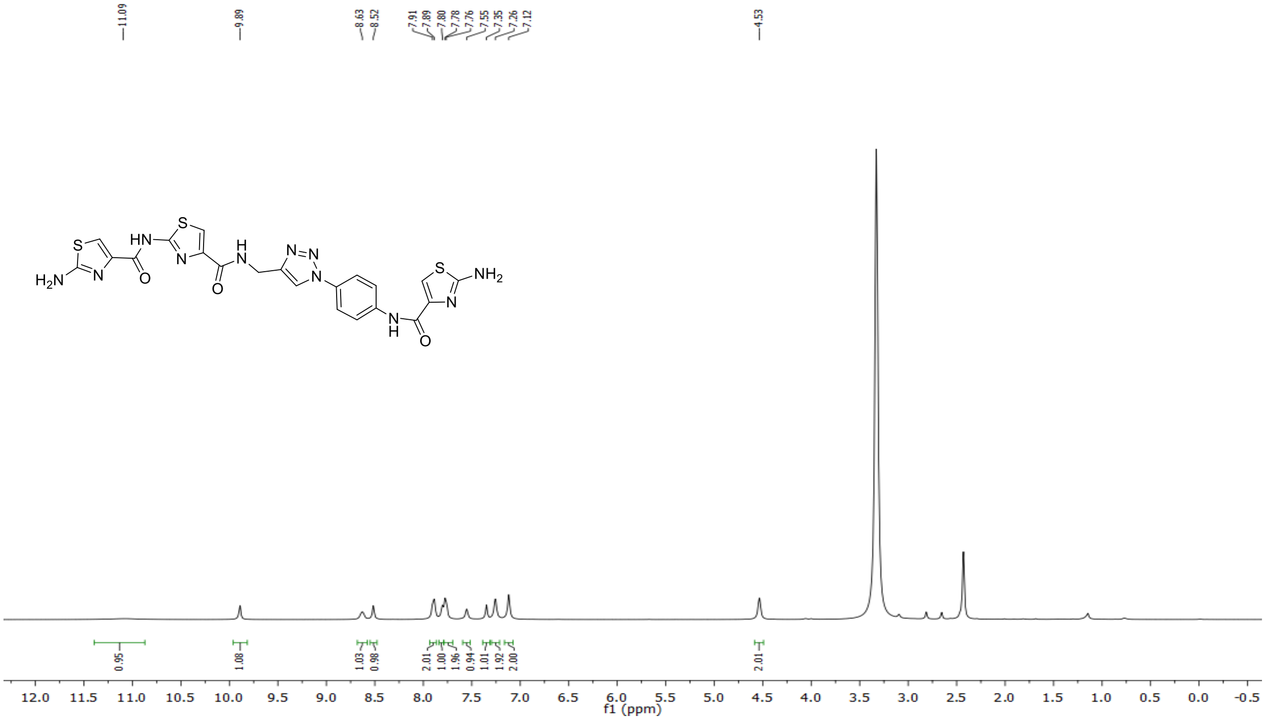

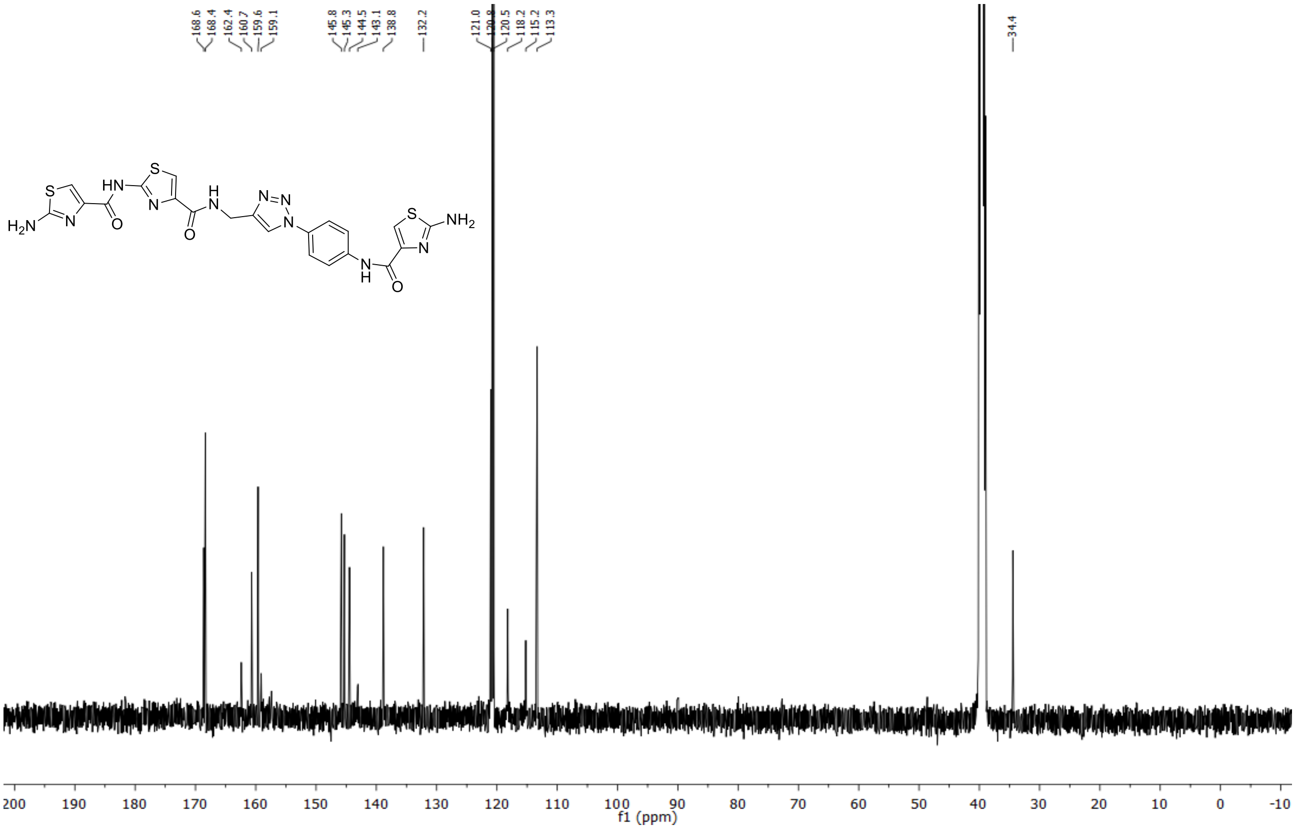

^{1}H and ^{13}C NMR of compound TTh2:

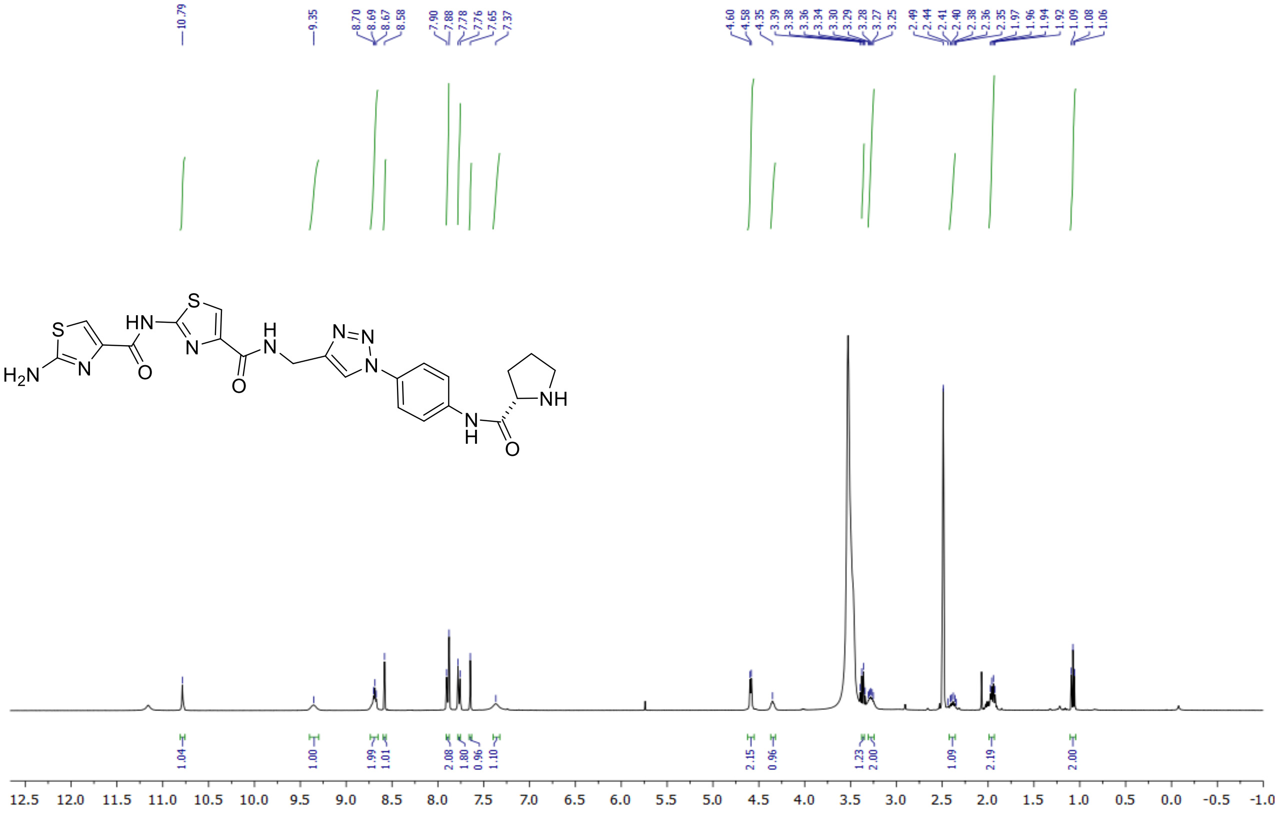

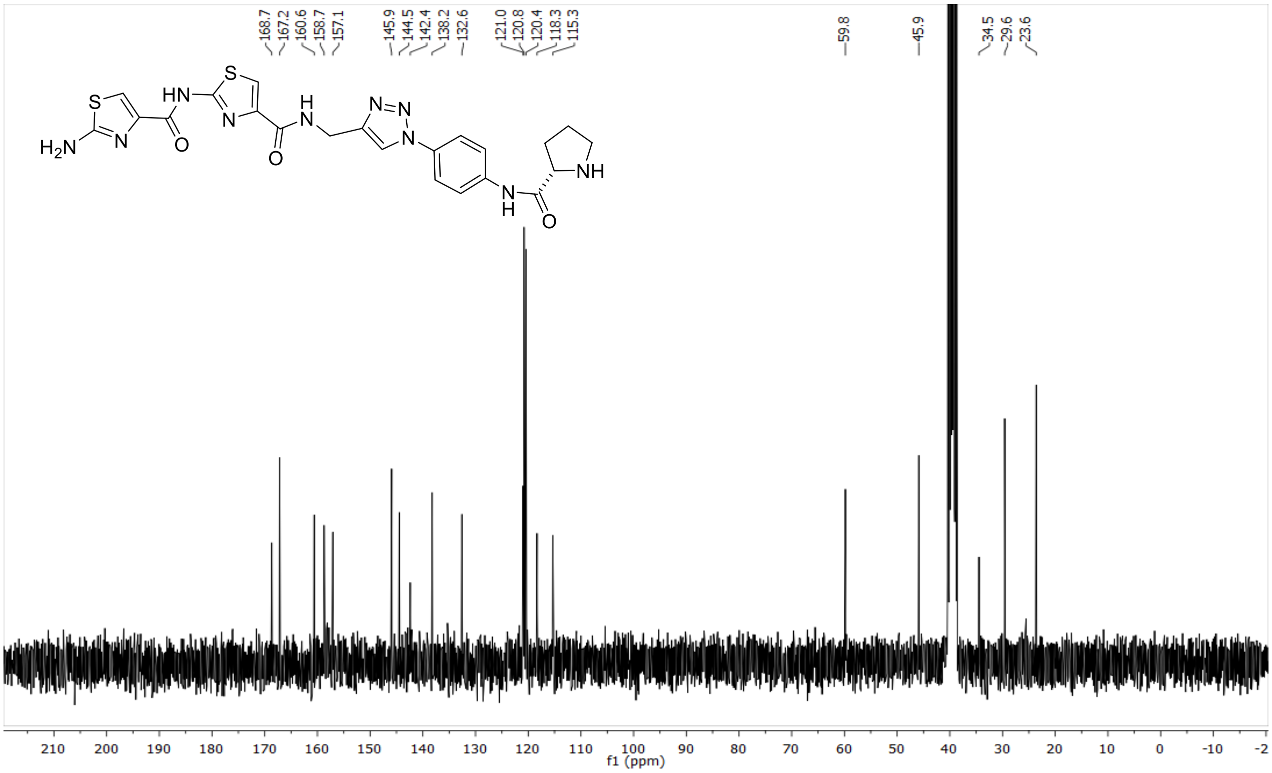

4.0 Mass analysis

Mass spectrum of S10

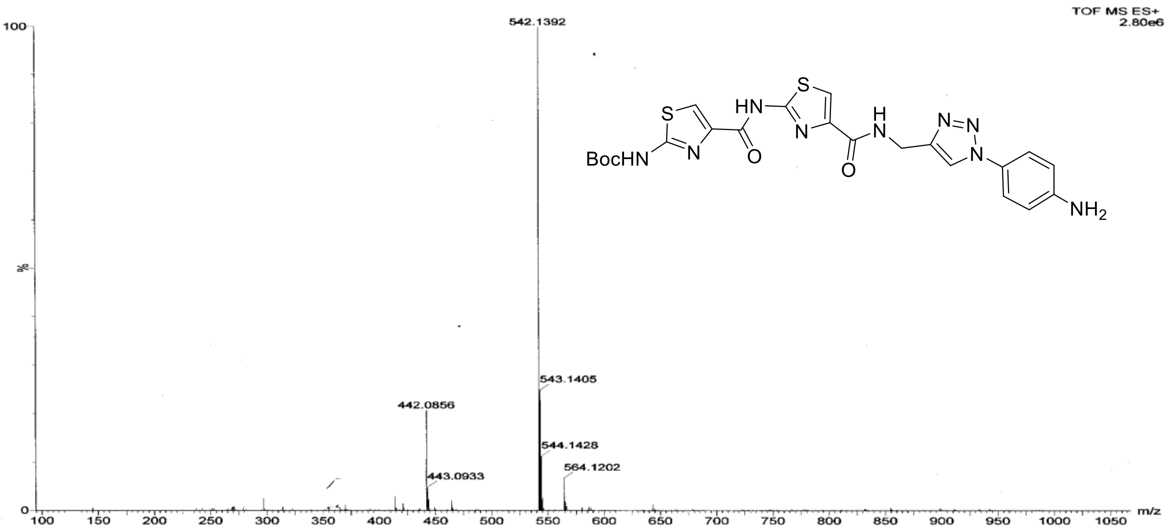

Mass spectrum of TTh1

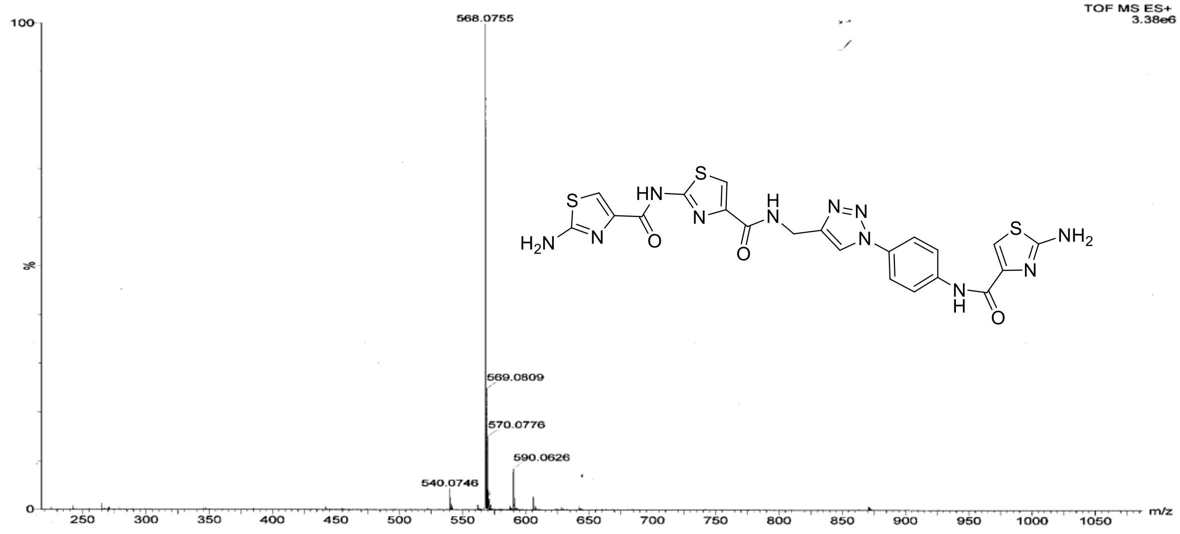

Mass spectrum of TTh2

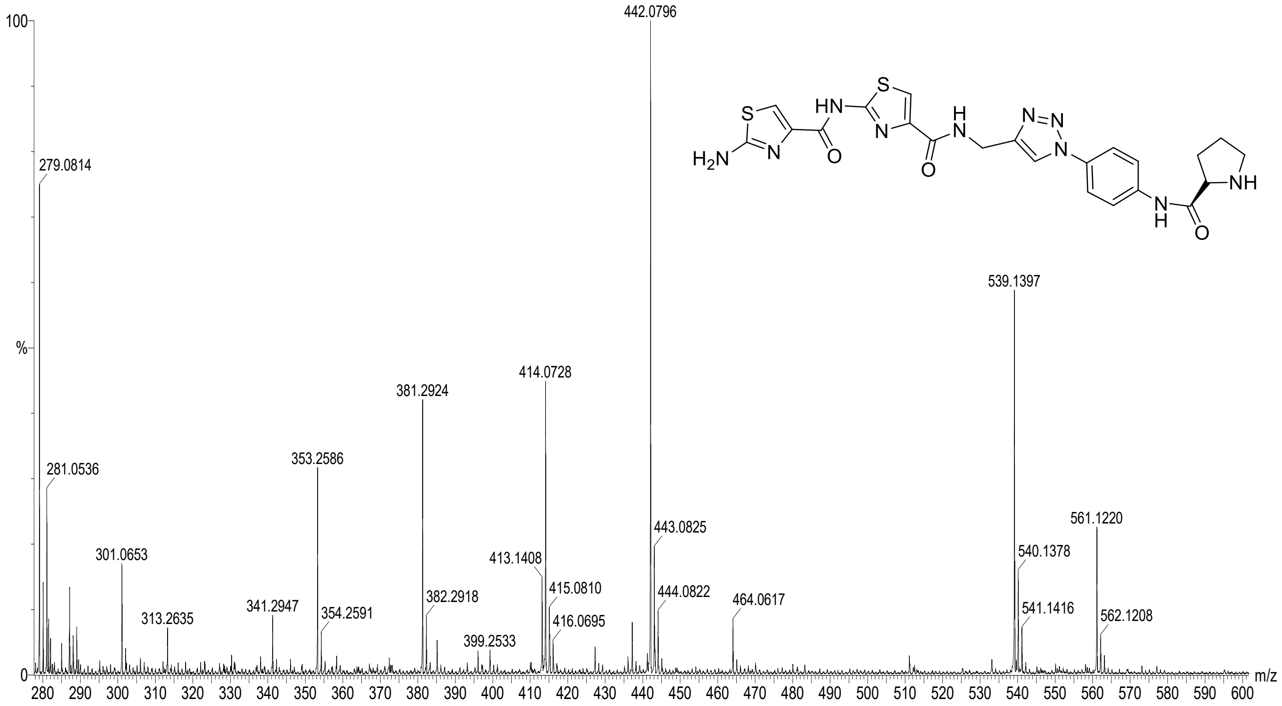

5.0 HPLC analysis.

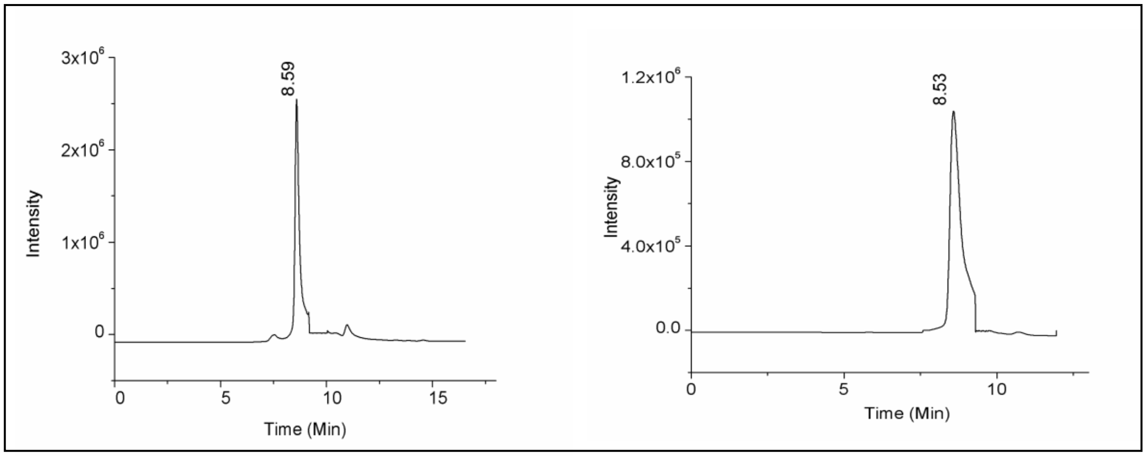

Figure S1. HPLC chromatograms of (A) **TTh1**; (B) **TTh2;** respectively.

6.0 UV-vis spectra

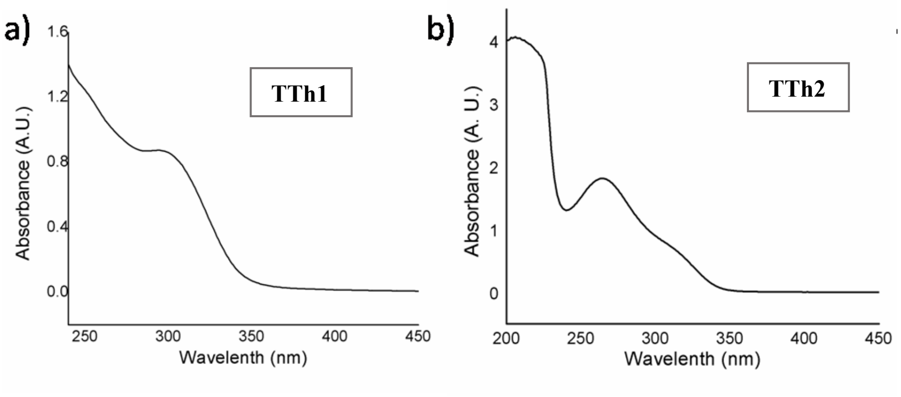

Figure S2. UV-visible spectra of a) **TTh1** and b) **TTh2,** respectively.

7.0 FRET melting assay

FRET melting assay using ligands **TTh1, TTh2** and **PhenDC3** were carried out in a 96-well format on a real-time PCR apparatus (Roche LightCycler® 480 II). Dual labeled DNA sequences with a donor fluorophore 6-carboxyfluorescein (5′ -FAM) and an acceptor fluorophore 6-carboxytetramethylrhodamine (3′ -TAMRA) were used for the study.

- *VEGF:* 5' FAM- d($CG_4CG_3C_2G_5CG_4T$)-TAMRA 3'
- *KRAS:* 5' FAM -d($AG_3CG_2TGTG_3A_2GAG_3A_2GAG_5AG_2$)- TAMRA 3'
- *c-KIT1:* 5' FAM- d($G_3AG_3CGCTG_3AG_3AG_3$)-TAMRA 3'
- *c-KIT2:* 5' FAM- d($G_3CG_3CGCGAG_3AG_4$)-TAMRA 3'
- *BCL2*: 5' FAM-d($G_3CGCG_3AG_2A_2T_2G_3CG_3$)-TAMRA 3'
- *c-MYC27:* 5' FAM -d($TGAG_3TG_3TAG_3TG_3TA_2$)- TAMRA 3'
- dsDNA: 5' FAM-d($CA_2TCG_2ATCGA_2T_2CGATC_2GAT_2G$)-TAMRA 3'

100 μM stock solutions of peptides **TTh1**, **TTh2 and PhenDC3** were prepared in 60 mM potassium cacodylate buffer, or 100 mM KCl, 10 mM Tris·HCl buffer, pH 7.4. Dual labeled oligos were diluted from stock to a concentration of 400 nM in the same buffer. The diluted samples were annealed by heating to 95° C for 5 min followed by gradual cooling at 25° C and incubated overnight at 4° C. Sample solutions were prepared in a 96-well plate (100 μl final volume) by mixing pre-annealed DNA (at 200 nM final concentration) with peptides (0.5, 1, 5 and 10 μM final concentration) in respective buffer (60 mM potassium cacodylate, or 100 mM

KCl, 10 mM Tris·HCl buffer, pH 7.4). After 1 h incubation, measurements were made in triplicate with excitation at 483 nm and detection at 533 nm. Final analysis of the data was carried out using OriginPro 2018 (OriginLabCorp.).

For competition experiments, pre-annealed, unlabeled calf thymus competitor (CT-DNA) at final concentrations of 1.0 and 10.0 μM was mixed to pre-annealed dual labeled quadruplex sequences (at 200 nM final concentration) in 60 mM potassium cacodylate buffer. Peptide **TTh2** was added into the DNA solution mixture at a final concentration of 1.0 μM. The DNA-peptide mixture was loaded in a, Roche 96-well plate and incubated for 1 h. Measurements were made in triplicate with excitation at 483 nm and detection at 533 nm using LightCycler® 480-II System (Roche). Final analysis of the data was carried out using OriginPro 2018 (OriginLab Corp.).

Table S1. FRET-melting analysis [ΔT_m (°C) values] of G4s and a control dsDNA in the presence of increasing amounts of (0 - 10 µM) thiazole peptides TTh1, TTh2 and PhenDC3

	ΔT_m (°C) [b]						
	VEGF [a]	*KRAS* [a]	*BCL2* [a]	*c-KIT1* [a]	*c-KIT2* [a]	*c-MYC27* [a]	dsDNA [a]
[**TTh1**] (µM)							
0.5	0	0.2	0	1.4	0	0	0
1	0	3	0	2.6	0.2	0	0
5	2.28	4.4	0	2.7	1.2	0	0
10	3.1	5	0	3.2	1.7	0	0
[**TTh2**] (µM)							
0.5	0.42	4.56	0	3.3	0.7	0	0
1	1.1	12.9	0	7.2	1.23	1	0.07
5	1.9	24.7	0	14	7.6	5.1	0.6
10	6.23	27.6	0	16	8.9	5.2	1
[**PhenDC3**]							
1	11	12.8	8.6	14.7	13.5	11.6	0.01
5	13	25	9.7	16.4	14.9	12.4	1.45
10	14	35.6	12	20.3	15.7	17.6	2.28

[(a)] The T_m values of the quadruplexes in 10 mM Tris-HCl buffer containing 60 mM KCl, pH 7.4 in the absence of ligands are: *VEGF* (76°C ±1), *KRAS* (50 °C ±1), *c-MYC27* (67 ±1), *BCL2* (74±1), *c-KIT1* (66 ±1), *c-KIT2* (70 ±1), *ds* DNA (73±1) ˚C; maximum measurable T_m = 94 ˚C. [(b)]ΔT_m at 1 µM ligand concentration [˚C] (ΔT_m = ± 1 ˚C).

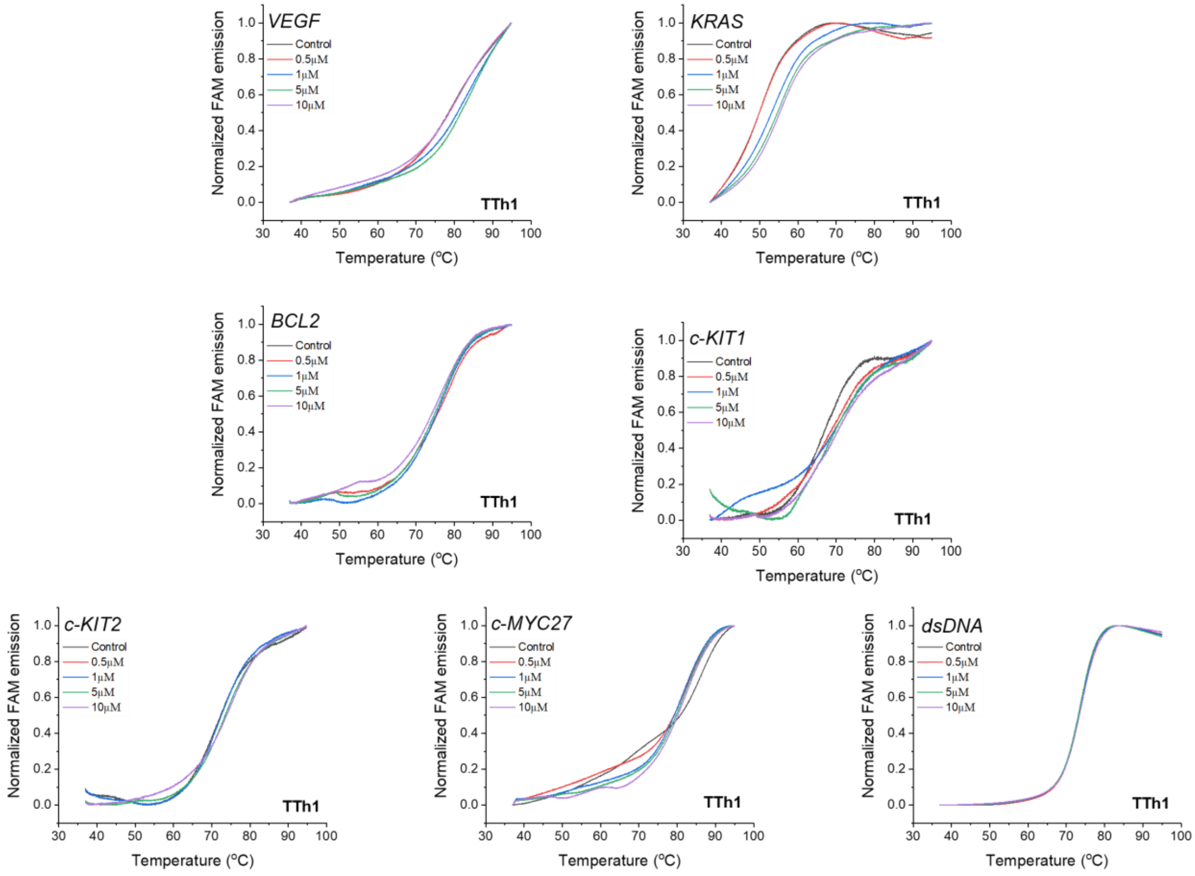

Figure S3. FRET-melting experiments performed with dual labeled G4 DNAs and dsDNA in the presence of **TTh1** (0-10 μM).

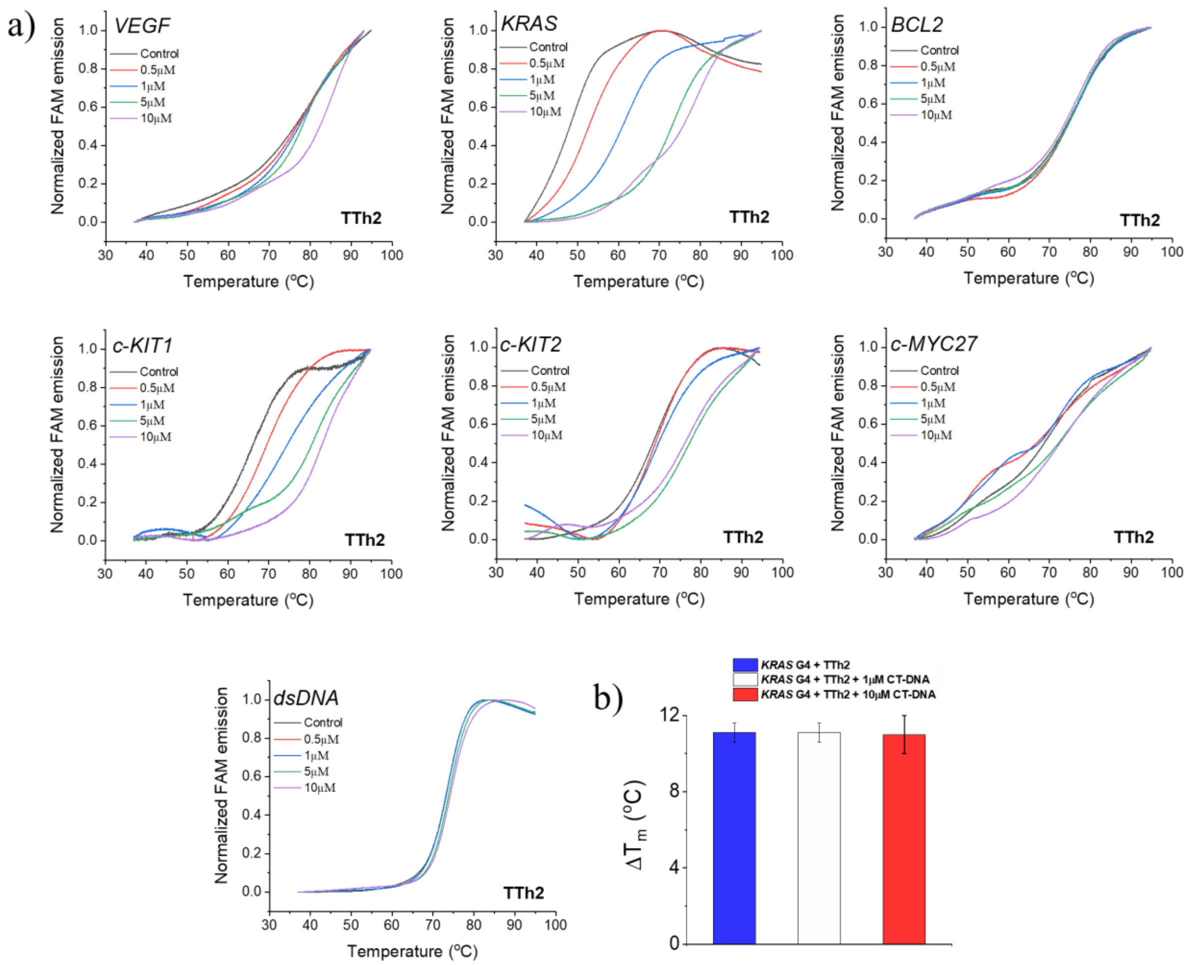

Figure S4. a) FRET-melting experiments performed with dual labeled G4 DNAs and dsDNA in the presence of **TTh2** (0-10 μM). b) Bar diagram showing no change in melting temperature (ΔT_m) of dual labeled *KRAS* G4 DNA in the presence of 1μM of **TTh2** upon addition of 1 and 10μM unlabeled CT-DNA.

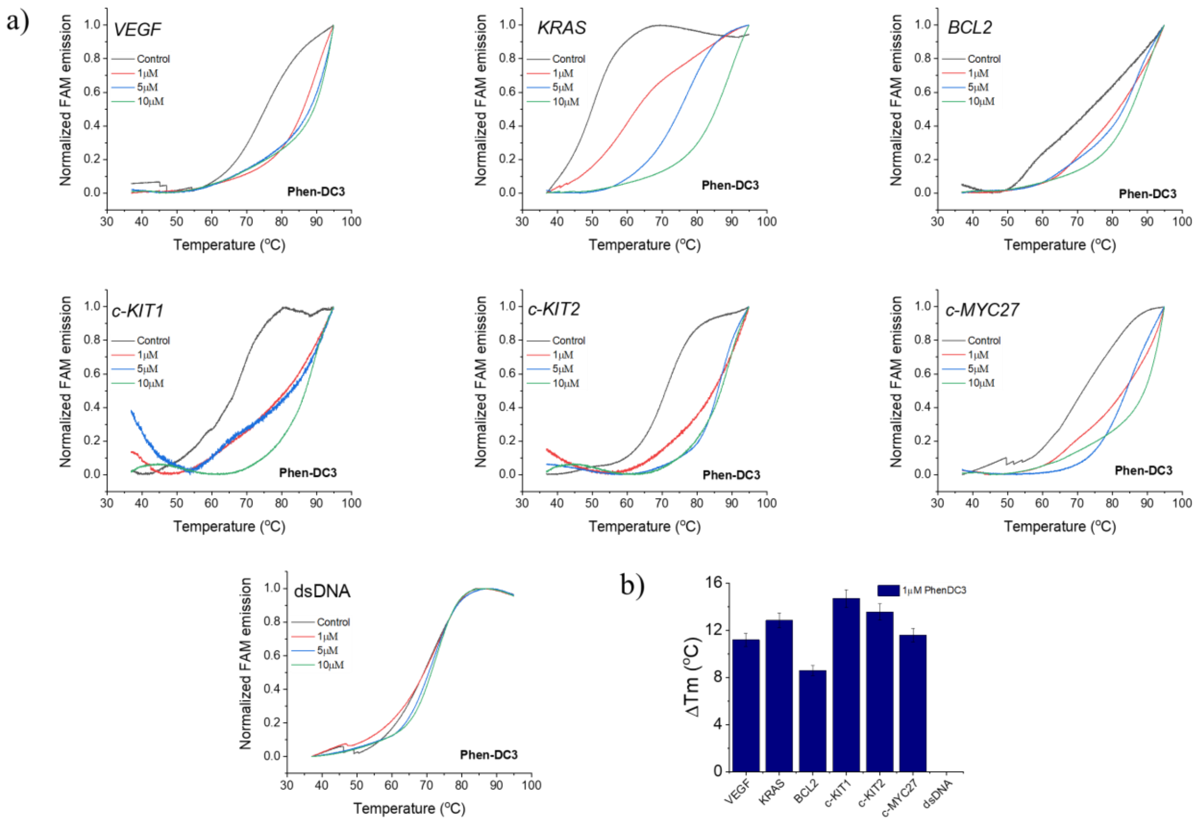

Figure S5. a) FRET-melting experiments performed with dual labeled G4 DNAs and dsDNA in the presence of **PhenDC3** (0-10 μM). b) Bar diagram showing remarkable change in melting temperature (ΔT_m) of dual labeled G4 DNAs in the presence of 1μM of **PhenDC3.**

8.0 Fluorometric titration

Thiazole peptides **(TTh1** and **TTh2)** were diluted in filtered and degassed Tris-KCl buffer (100 mmol Tris.KCl buffer, pH~7.4) to a final concentration of 1 μM. Fluorescence titrations were performed with successive addition of pre-annealed DNA into the solution. The fluorescence spectra were recorded on a Horiba Jobin Yvon Fluoromax 3 instrument at 25°C in a 10mm path-

length quartz cuvette with filtered 100 mM Tris-KCl buffer (pH~7.4). The DNA sequences (5′→3′) used in fluorimetric titrations are as follows:

- *VEGF:* 5’ $CG_4CG_3C_2G_5CG_4T$ 3’
- *KRAS:* 5' $AG_3CG_2TGTG_3A_2GAG_3A_2GAG_5AG_2$ 3'
- *BCL2*: 5' $G_3CGCG_3AG_2A_2T_2G_3CG_3$ 3'
- *c-KIT1:* 5’ $G_3AG_3CGCTG_3AG_3AG_3$ 3’
- *c-KIT2:* 5’ $G_3CG_3CGCGAG_3AG_4$ 3’
- *c-MYC27:* 5' $TGAG_3TG_3TAG_3TG_3TA_2$ 3'
- *dsDNA*: 5' $CA_2TCG_2ATCGA_2T_2CGATC_2GAT_2G$ 3'

F is the fluorescence intensity, F_{max} is the maximum fluorescence intensity, F_0 is the fluorescence intensity in the absence of DNA and K_d is the dissociation constant. All the binding constants of fluorescence spectral data has been calculated using the following Hill 1 equation with the help of OriginPro 2018:

$$F = F_0 + \frac{(F_{max} - F_0)[DNA]}{K_d + [DNA]}$$

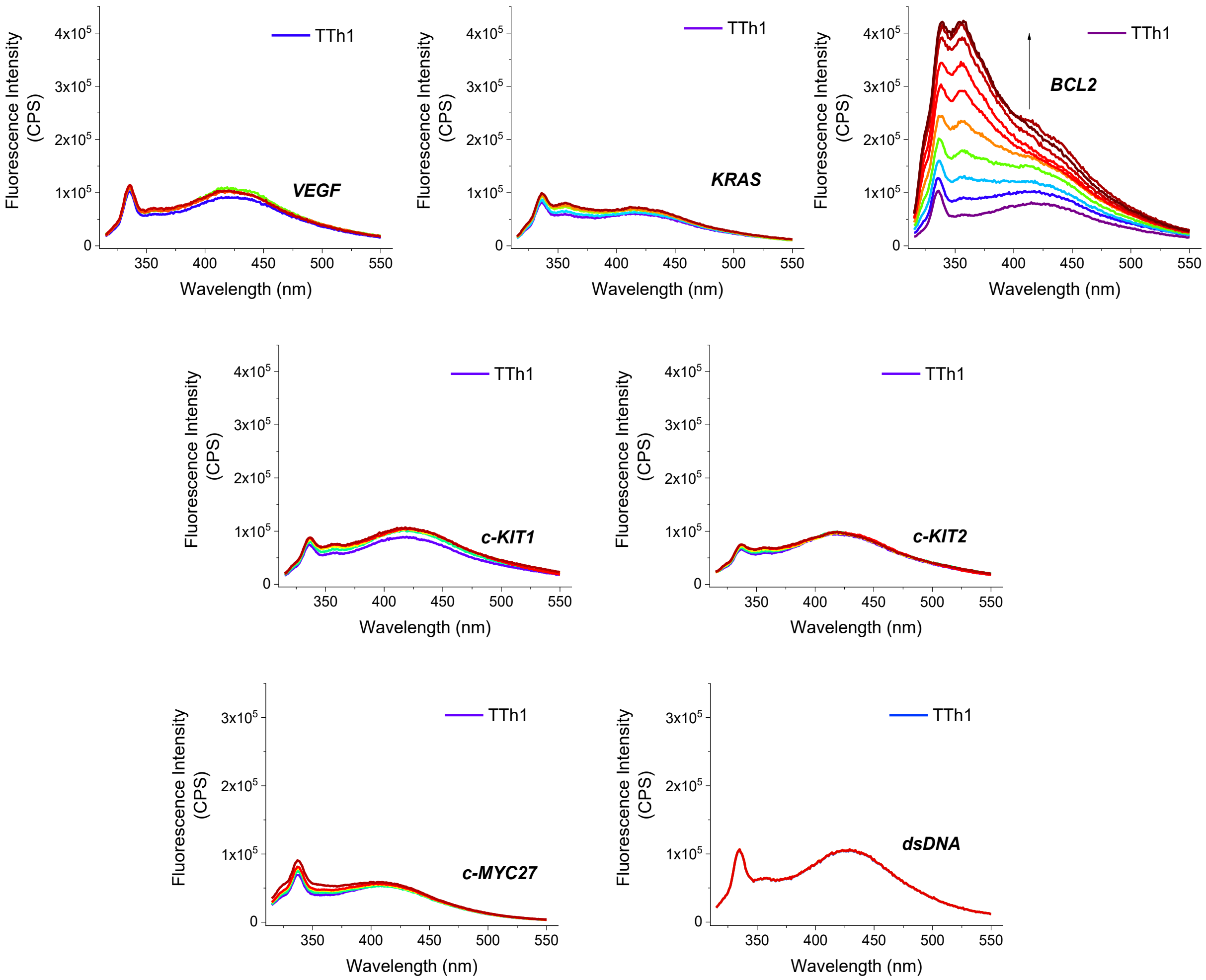

Figure S6. Fluorescence titration of 2 μM of **TTh1** upon addition of DNA structures in 100 mM Tris.KCL buffer, pH~7.4.

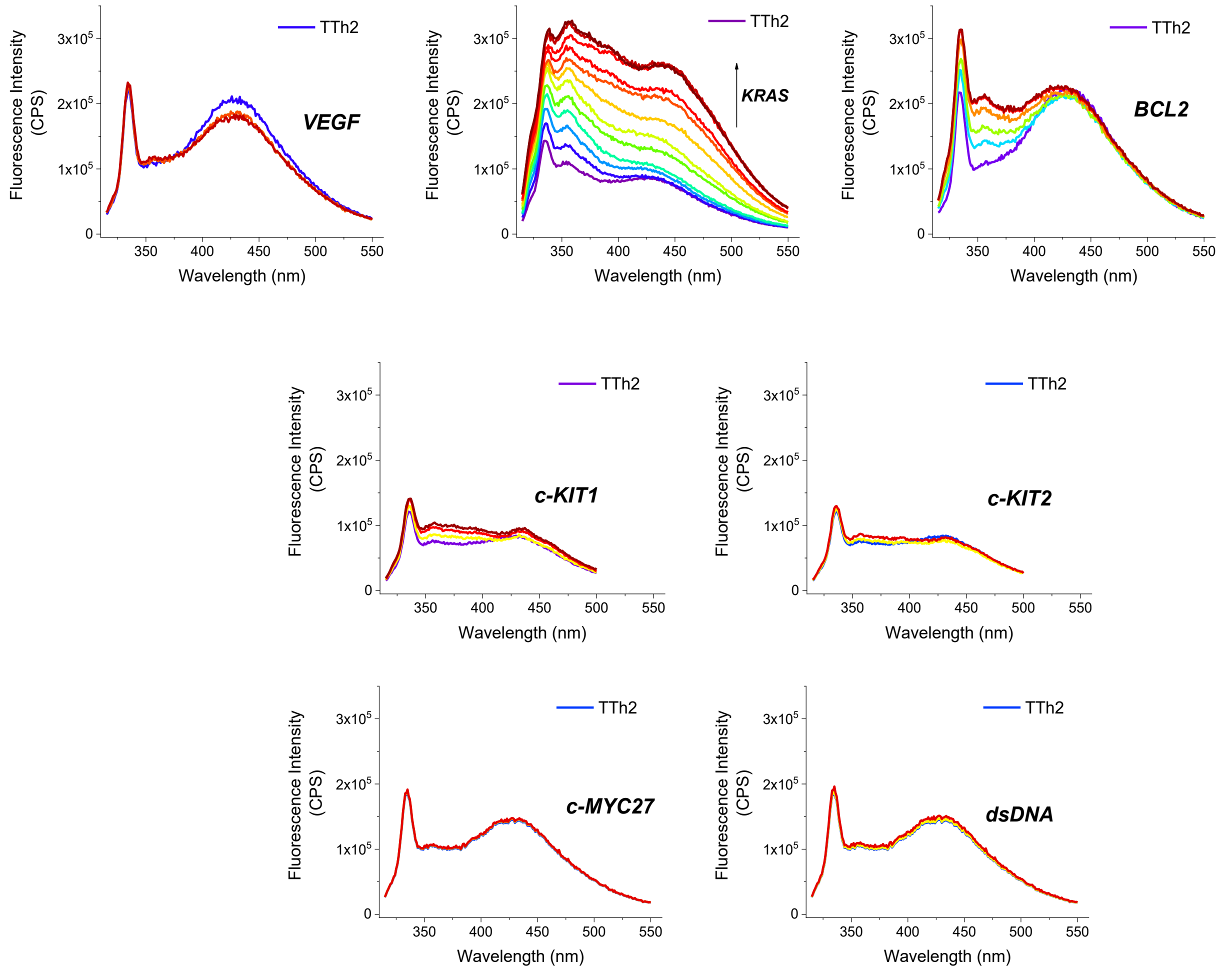

Figure S7. Fluorescence titration of 2 μM of **TTh2** upon addition of DNA structures in 100 mM Tris.KCL buffer, pH~7.4

9.0 Isothermal calorimetry

Isothermal titration calorimetric experiments were performed with a MicroCal PEAQ-ITC apparatus, Malvern Instruments (Great Britain). Titrations were carried out at a constant temperature of 25 °C, with a reference power set to 10 µcal s^{-1}. Quadruplex solutions prepared in 100 mM Tris-KCl underwent a slow cooling before each experiment. The sample cell was filled with a 5 µM solution of DNA, and the reference cell contained 100 mM Tris-KCl. A 50 µM ligand solution was micro-injected from a computer-controlled microsyringe into the sample cell. All solutions were degassed for a few minutes to get rid of air bubbles. The first injection (0.4 µL) was excluded from the analysis to avoid artifacts because it may have been affected by diffusion during the equilibration phase, which may change the local concentration of quadruplex at the position near the needle of the syringe. Baseline stability was achieved before the first injection. Molar binding enthalpies were calculated by integration of the power output after each injection, normalized to the moles of ligand added, and corrected for dilution heats measured by titrating ligand into buffer in the same experimental conditions. The data was analyzed by MicroCal PEAQ-ITC analysis software. Thermograms, corrected for dilution heats, were fitted in order to obtain thermodynamic parameters, which are provided in Table S2.

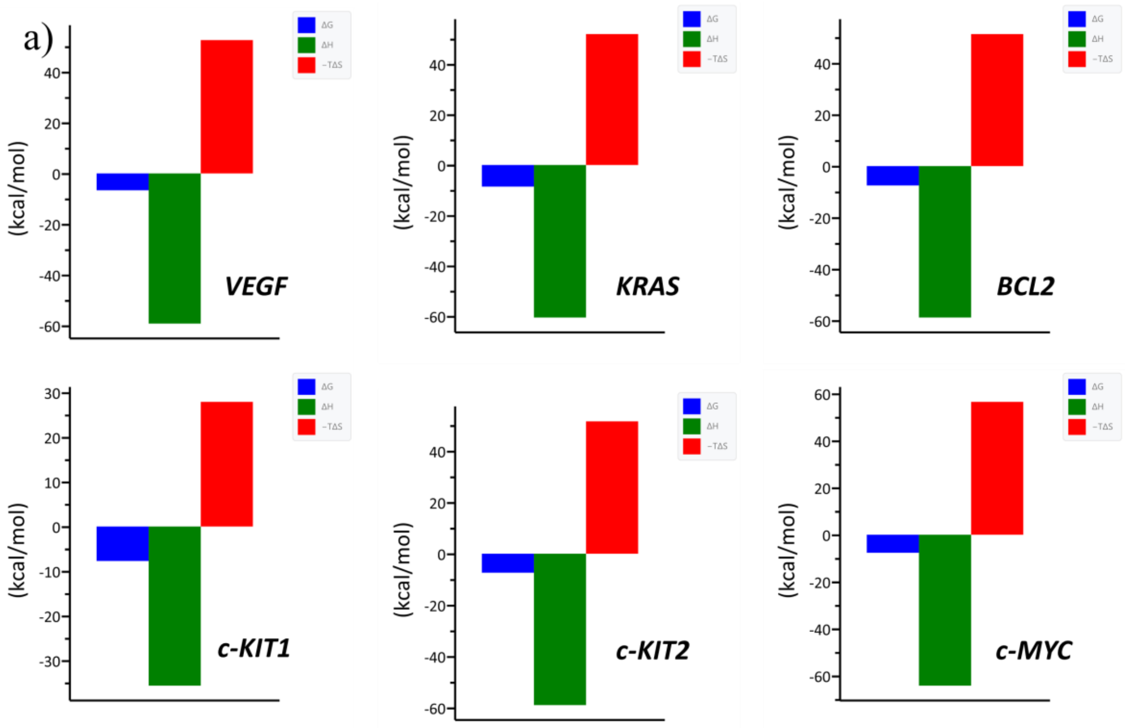

b)

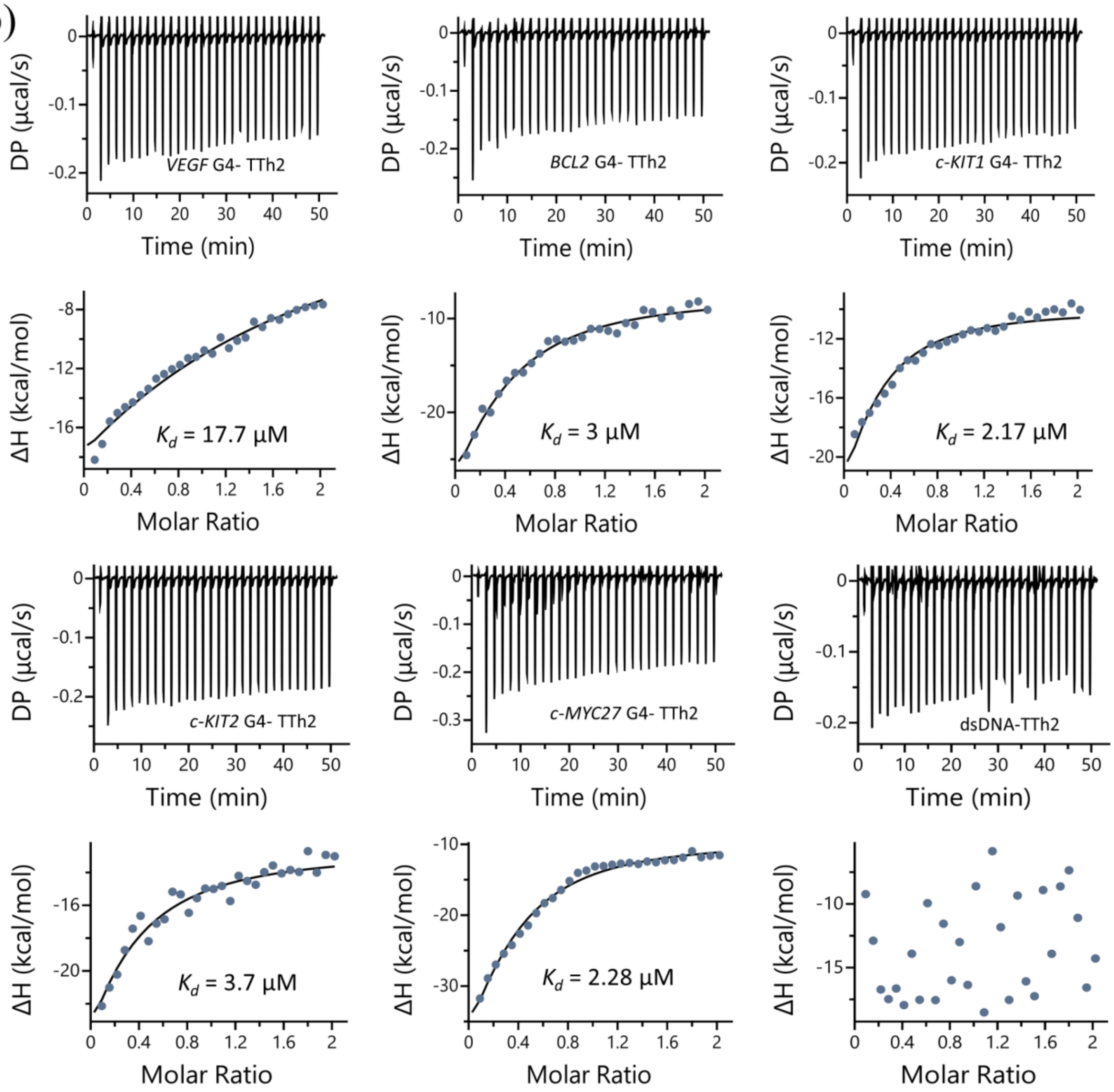

Figure S8. a) Signature plot showing ΔG, ΔH and TΔS of binding interactions of G4-**TTh2.** b) Isothermal titration calorimetry analyses for the binding of **TTh2** (50 µM) with G4s (5 µM).

Table S2. Summary of stabilization potential (ΔT_m), binding affinity (K_d) and thermodynamic properties of ligand TTh2 for different G-quadruplexes and duplex DNA.

G-quadruplex	ΔT_m (°C) at 1 µM ligand concentration	K_D (µM) Fluorescence Spectroscopy	n	K_D (µM) ITC	ΔG (kcal.mol^{-1})	ΔH (kcal.mol^{-1})
VEGF	1.1	ND	1	17.7 ± 4	-6.5 ± 0.1	-59.2 ± 5.5
KRAS	**12.9**	**0.8**	**1**	**0.53± 0.25**	**-8.56 ± 0.4**	**-60.5 ± 10**
BCL2	0	1.5	1	3 ± 0.1	-7.5 ± 0.04	-59 ± 9
c-KIT1	7.2	2	1	2.2 ± 1	-7.7 ± 0.5	-35.6 ± 13.6
c-KIT2	1.23	1.5	1	3.7 ± 0.3	-7.4 ± 0.05	-59 ± 8.5
c-MYC27	1	2.5	1	2.28±0.74	-7.7 ± 0.23	-64.2 ± 4
dsDNA	0.07 ± 0.01	2.2	ND	ND	ND	ND

10.0 Circular Dichroism spectroscopy

CD titrations were performed in Jasco J815 spectropolarimeter using quartz cuvette of path length 1 mm at 25 °C. All the spectra were recorded in the wavelength range of 200−400 nm at a scan rate of 200 nm/min and averaged over three scans. A buffer baseline was collected in the same cuvette and was subtracted from the sample spectra. DNA sequences were pre-annealed in Tris-KCl buffer (100 mM KCl, 10 mM Tris•HCl, pH 7.4) by heating in a dry bath to 95 °C for 5 min followed by cooling at 25 °C and incubated overnight at 4 °C. CD titration assay was performed at a fixed concentration of 15 µM of *KRAS* G-quadruplex. The spectra were recorded after each successive addition of ligand **(TTh2)** into the *KRAS* G-quadruplex solution. The final analysis of the recorded spectra was conducted using OriginPro 2018 (OriginLab Corp.).

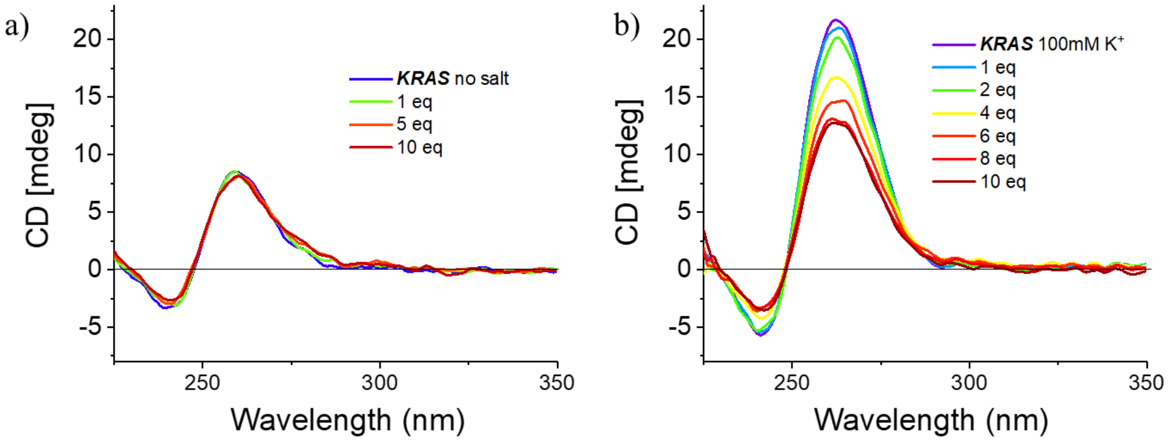

Figure S9. CD spectra of 15 μM *KRAS* in Tris buffer (pH 7.4), in the absence (a) and presence (b) of added salt (100mM KCl), titrated with 0-10 equiv of **TTh2**.

11.0 Molecular docking and molecular dynamics simulation studies

Molecular Docking

The NMR structure (PDB id: 6T2G) of 32 mer *KRAS* G-quadruplex DNA has been retrieved from the RCSB protein data bank. The monomeric unit (A chain) has been used in the docking studies. The 'Prepare Protein' protocol of MGL tools has been executed for inserting hydrogen in the nucleic acids of the G-quadruplex DNA structure, modeling missing loop regions, and removing water and other heteroatoms from the G-quadruplex DNA structure (1). Molecular docking has been performed with auto dock vina (2).

The docking procedure produced 20 different conformers. The best conformers were selected based on the binding free energy and visual inspection and further analyzed. After docking, it was observed that **TTh2** belongs to the category of DNA groove binder. **TTh2** shows a good binding affinity toward DNA with a docking score of -9.5 kcal/mol. It has been observed that **TTh2** interacts with the Guanine (dG4, dT8, dG26, dG28), and adenine (dA1) of *KRAS* DNA in the parallel folded Guanine region by forming eleven strong hydrogen bonding in the range of 1.84724 angstrom (Å) to 3.22443angstrom (Table S3). It has also formed four pi bonds with G-quadruplex DNA. The eleven hydrogen bonds support the tight binding of the **TTh2** with *KRAS* G-quadruplex DNA.

The contribution of the proline moiety of the **TTh2** ligand was important in binding by creating three essential hydrogen bonds with the *KRAS* G-quadruplex DNA (Table S3). The Guanine 4, Guanine 28, and Thymine 8 are the three of them that suggest that proline has a crucial function to hold the ligand in place within the DNA structure. Because of its rigid cyclic nature, proline imposes conformational restraints that predispose to minor groove binding, causing greater spatial complementarity between ligand and DNA surface. Proline-substituted ligands have also been observed to be more resistant to enzymatic attack by virtue of the steric bulk brought into the molecule by the proline ring which attribute in enhancing ligand stability and bioavailability in the biological milieu.

Molecular Docking with other G4 DNAs

We performed molecular docking of **TTh2** with other G4s (like *BCL2* and *cMYC*) and found that while the **TTh2** ligands were capable of binding to these alternative G4 structures, the binding affinities, as reflected by the docking scores, were consistently less favorable compared to the *KRAS* G4. Specifically, the docking scores (in kcal/mol) for the ligands against multiple G4 targets are summarized as follows:

G4 Target	Docking Score (kcal/mol)	Binding Mode Observed
KRAS (PDB:6T2G)	−9.5	Groove binding interactions, strong stabilization
BCL2 (PDB:2F8U)	−6.2	Weaker binding, limited groove complementarity
c-MYC (PDB:2L7V)	−7.6	Moderate binding, less optimal groove fit

Notably, the *KRAS* G4 structure provided the most favorable docking scores, indicating stronger predicted binding affinity. This is likely due to the unique groove architecture and loop

arrangements of the *KRAS* G4, which better complement the structural features of the **TTh2** ligands, such as their planar aromatic systems and flexible side chains capable of engaging in hydrogen bonding and π–π stacking interactions.

In contrast, the *c-MYC* and *BCL2* G4 structures exhibited less pronounced groove complementarity with the ligands, resulting in binding poses characterized by fewer stabilizing contacts. For example, the hydrogen bonding networks and π–π stacking interactions, critical for effective G4 stabilization, were less extensive in these alternative G4 complexes. This difference in interaction quality is reflected in the relatively higher (less favorable) docking scores and suggests reduced ligand efficacy at these sites.

Moreover, the *KRAS* G4 loops and grooves may provide more accessible binding pockets for the **TTh2** compounds, enhancing both binding specificity and stability. Such structural compatibility is crucial for effective G4 targeting, especially in the context of anticancer drug design, where selectivity can reduce off-target effects.

Taken together, these findings support the preferential affinity of the **TTh2** ligands toward the *KRAS* G4 structure. Consequently, our detailed molecular modeling and simulation studies have been focused on the *KRAS* G4, as it represents the biologically relevant and most promising target for the ligands investigated.

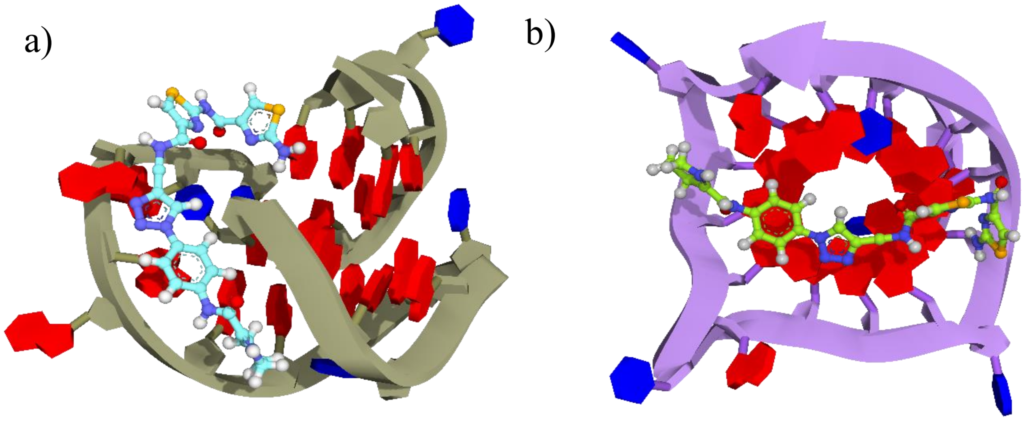

Figure S13. a) Interaction between the proline part of **TTh2** with *BCL2* G-quadruplex. b) Interaction between the proline part of **TTh2** with *c-MYC* G-quadruplex

Molecular Dynamics Simulation

To gain a deeper understanding of the structural stability and conformational dynamics of the DNA (*KRAS*)-ligand (**TTh2**) complex, extensive molecular dynamics (MD) simulations were performed. MD simulations were executed using NAMD, employing the CHARMM36m force field parameters for nucleic acids and ions (5). The SwissParam server was used to generate the topology and parameter files for the best-docked ligand (6), enabling its integration into the DNA system with consistent force field treatment.

The docked DNA-ligand complex was solvated in a periodic truncated octahedral water box, maintaining a minimum padding of 10 Å between the solute and the box edge to prevent edge effects and ensure adequate solvation. To mimic physiological ionic strength and maintain electroneutrality, sodium (Na^+) and chloride (Cl^-) ions were added to achieve a final salt concentration of 0.15 M. Ionization was performed using the Autoionize plugin in VMD. The final solvated and neutralized system contained DNA, the ligand, water molecules, and counterions. Following system construction, energy minimization was performed for 9,000,000 steps, corresponding to approximately 45 nanoseconds. The periodic boundary dimensions were set as follows: X: 31.616, Y: 13.438, Z: 14.235. During equilibration, positional restraints were applied to both DNA and the ligand. This prolonged minimization helped relieve steric clashes, relax the initial geometry, and optimize solvent organization around the solute. The system was then gradually equilibrated using a multi-step protocol. Initially, equilibration was conducted under the NVT ensemble (constant number of particles, volume, and temperature), followed by the NPT ensemble (constant pressure and temperature). Throughout the equilibration, harmonic positional restraints were applied to the heavy atoms of both DNA and ligand to preserve the initial binding conformation while allowing the solvent and ions to equilibrate freely. Long-range electrostatic interactions were calculated using the Particle Mesh Ewald (PME) method (7).

The production MD simulation was conducted for 200 nanoseconds without any restraints. A 2-femtosecond integration time step was used, with all covalent bonds involving hydrogen atoms constrained. Coordinates, velocities, and energies were saved every 2 picoseconds. Trajectory analysis was carried out using VMD 1.9.3 and Python scripts.

Molecular dynamics simulation

For a clearer understanding of stability and dynamic performance of the DNA-ligand complex, we carried out a 200 ns molecular dynamics (MD) simulation of *KRAS* DNA in complex with **TTh2**. The simulation was designed to validate docking interactions, probe conformational stability, and investigate long-term persistence of significant non-covalent interactions.

RMSD

Root Mean Square Deviation (RMSD) is a widely used metric to evaluate the average positional deviation of atoms from a reference structure, offering insight into the overall structural stability of a molecular system during simulation (8). We have calculated the root mean square deviation (RMSD) of the trajectories to observe the fluctuations of the ligand (**TTh2**) and DNA backbone atoms and the stability of the simulated system. The DNA RMSD profile (figure 2a) showed a rapid increase during the initial equilibration phase (0–40 ns), rising from approximately 0.35 nm to ~0.75–0.80 nm. This initial rise is characteristic of structural relaxation from the energy-minimized starting conformation to a more energetically favorable dynamic state. After ~40 ns, the system began to plateau, indicating that the G-quadruplex had reached a stable conformational state.Throughout the remainder of the simulation (40–200 ns), the RMSD values fluctuated within a narrow range of ~0.75 to 0.85 nm, without any signs of structural drift, collapse, or unfolding. This behavior suggests that the G-quadruplex fold was well maintained under the simulated conditions. The stable RMSD indicates that the G-quadruplex structure remains intact and is not destabilized by ligand binding.

We calculated the RMSD of the **TTh2** ligand over 200 ns to assess its conformational stability during binding with the *KRAS* G-quadruplex DNA (Figure 2b). RMSD is commonly used to evaluate how much a molecule deviates from its initial position over time, providing insight into binding stability and structural adaptation.The **TTh2** ligand exhibited an initial increase in RMSD during the first 30 ns, rising from approximately 0.08 nm to ~0.25–0.30 nm. This early fluctuation reflects conformational adjustment as the ligand relaxes from its docked pose and adopts a more favorable orientation within the G-quadruplex environment.Following this equilibration phase, the ligand RMSD stabilized and fluctuated within a narrow range of ~0.18 to

0.25 nm for the remainder of the trajectory. These low and consistent RMSD values indicate that **TTh2** remained firmly bound within the G-quadruplex binding pocket or groove and did not undergo any significant conformational rearrangement or unbinding events.The absence of large deviations or structural drift suggests that the ligand adopts a stable and persistent binding mode, which supports its potential role as a G-quadruplex-targeting agent. These findings are in agreement with the DNA RMSD profile, further confirming that ligand binding does not destabilize the quadruplex structure but instead maintains a well-defined interaction throughout the simulation.

RMSF

Root Mean Square Fluctuation (RMSF) is used in molecular dynamics simulations to assess the residue-level flexibility of a biomolecule by measuring the time-averaged deviation of each atom from its mean position (9). It provides critical insight into which regions of a structure are rigid or flexible, helping to identify stabilized binding sites, dynamic loops, or conformational hotspots influenced by ligand interaction or solvent exposure. In the context of the *KRAS*–**TTh2** interaction, RMSF provided insight into how **TTh2** binding contributes to the conformational rigidity of the G-quadruplex core, particularly at the interaction interface, while allowing the loop and terminal regions to retain their natural flexibility.

To evaluate the residue-level flexibility of the *KRAS* G-quadruplex DNA in complex with the **TTh2** ligand, we performed Root Mean Square Fluctuation (RMSF) analysis over a 200 ns MD trajectory (Figure 2c). RMSF quantifies the time-averaged fluctuation of each atom or residue from its mean position, offering valuable insights into regions of structural rigidity and local flexibility. This is especially important for non-canonical DNA structures like G-quadruplexes, where loop dynamics and tetrad integrity influence both folding and ligand recognition.The RMSF plot revealed that the majority of the central DNA residues-presumably corresponding to guanines involved in G-quartet stacking—exhibited low fluctuations in the range of 0.15–0.30 nm. This indicates that the core G4 scaffold remained conformationally stable throughout the simulation. The reduced flexibility in these regions suggests effective maintenance of Hoogsteen hydrogen bonding and π–π stacking interactions, which are essential for the structural integrity of G-quadruplexes. In contrast, increased fluctuations were observed at the terminal regions and

within loop segments, with RMSF values rising to ~0.4–0.85 nm. These peaks are attributed to unstacked or single-stranded guanine residues and looped-out nucleotides, which are naturally more exposed to solvent and not directly involved in G-tetrad formation. Such flexibility is typical for G4 structures and does not indicate destabilization, but rather reflects the intrinsic dynamic nature of loop and overhang regions.Notably, residues adjacent to the ligand binding site exhibited reduced flexibility compared to surrounding areas, suggesting local stabilization induced by **TTh2** binding. The rigidity observed in these segments implies that **TTh2** forms stable contacts,possibly hydrogen bonding or groove occupancy,that restrict atomic fluctuations in its immediate vicinity. This is consistent with the ligand and DNA RMSD data, which show overall conformational stability of the complex.

The radius of gyration (Rg)

The radius of gyration (Rg) is used in MD simulations to quantify the overall compactness and spatial distribution of a biomolecular structure around its center of mass. It provides insight into how tightly or loosely the atoms are packed, and is especially useful for monitoring global conformational changes such as folding, unfolding, compaction, or expansion during the simulation(10). In the context of DNA-ligand complexes, a stable Rg value indicates that the system maintains its structural integrity, whereas significant deviations may reflect disruption or instability in the molecular architecture.In the context of G-quadruplex DNA, Rg serves as an indicator of whether the folded topology is maintained or disrupted under ligand-bound conditions.

In this study, we have calculated Rg to evaluate whether the *KRAS* G-quadruplex DNA retained its structural compactness upon binding with the **TTh2** ligand over a 200 ns MD simulation. As presented in Figure 2d, the Rg of the DNA backbone exhibited a rapid decrease during the initial equilibration phase (0–30 ns), likely corresponding to system relaxation and stabilization from the energy-minimized starting structure. Following this period, the Rg stabilized and fluctuated consistently around ~1.4 nm, with only minor deviations observed throughout the remaining simulation time.This steady Rg profile indicates that the quadruplex retained its compact, folded architecture in the presence of **TTh2**, with no evidence of significant expansion, unwinding, or conformational collapse. Importantly, the Rg remained below 1.45 nm, which is typical for a

well-formed G-quadruplex core, suggesting that the Hoogsteen hydrogen bonding network and π–π stacking interactions among guanine tetrads remained intact.The absence of large-scale structural rearrangement suggests that **TTh2** binding does not disrupt the global folding of the G-quadruplex, a crucial property for any potential G4-targeting ligand. In fact, the consistent Rg values support the idea that **TTh2** may contribute to stabilizing the G4 conformation, either through groove binding or terminal stacking interactions. These findings are further supported by RMSD and RMSF analyses, which also indicate structural stability in the ligand-bound state.

Hydrogen Bond Analysis

Hydrogen bonding plays a central role in stabilizing biomolecular complexes and is frequently analyzed in molecular dynamics (MD) simulations to assess interaction strength, binding specificity, and complex stability over time (11). In the context of ligand–DNA systems, hydrogen bonds can indicate how tightly and persistently the ligand remains associated with its target, especially when other non-covalent interactions (e.g., π–π stacking or electrostatics) are also involved.In our study, we tracked the number of hydrogen bonds formed between the **TTh2** ligand and the *KRAS* G-quadruplex DNA throughout the 200 ns simulation (Figure 3e).

To assess the stability and interaction strength of the *KRAS* G-quadruplex DNA-**TTh2** complex, hydrogen bonding patterns were analyzed over a 200 ns molecular dynamics simulation (Figure Sx). The number of hydrogen bonds fluctuated between 0 and 5 throughout the trajectory, indicating dynamic yet persistent interactions between the ligand and the DNA. In the initial 0–50 ns phase, the complex maintained 1–2 hydrogen bonds intermittently, suggesting an early binding conformation with limited stabilization. As the simulation progressed, particularly after ~130 ns, a more consistent and higher number of hydrogen bonds (3–4 on average) was observed, pointing toward an increasingly stable interaction mode. The formation of up to five hydrogen bonds in certain intervals implies the involvement of key donor and acceptor groups in the ligand and G-quadruplex grooves or loops. This shift toward enhanced hydrogen bonding in the latter phase likely reflects the ligand's adaptation into a more favorable binding orientation, potentially stabilizing within the grooves of the G-quadruplex. These findings highlight the role of hydrogen bonding in mediating ligand recognition and anchoring, contributing significantly to the overall stability of the complex.The key DNA residues involved in hydrogen bonding with

TTh2 were identified as dG4, dT8, dG26, dG28, and dA1-regions associated with minor groove and G-quartet interfaces, which are often favorable for small molecule binding. These interactions likely contribute to the overall conformational stability of the complex, as supported by the RMSD, RMSF, and Rg analyses. The 200 ns molecular dynamics simulation of the *KRAS* G-quadruplex DNA in complex with the **TTh2** ligand revealed a structurally stable and well-maintained G4 architecture throughout the trajectory. The RMSD analysis demonstrated that both the DNA backbone and the **TTh2** ligand reached equilibrium early in the simulation and remained stable with minimal fluctuations, indicating that no major structural deviations or dissociation events occurred.The RMSF analysis highlighted that central guanine residues involved in G-quartet formation exhibited low flexibility, while loop and terminal regions remained relatively dynamic, as expected. Importantly, regions directly interacting with **TTh2** showed reduced atomic fluctuations, suggesting local stabilization induced by ligand binding.The radius of gyration (Rg) further confirmed the compactness and structural integrity of the G-quadruplex. After initial equilibration, the Rg values stabilized around 1.4 nm, reflecting a well-folded conformation preserved in the presence of the ligand. No signs of large-scale unwinding or expansion were observed. Hydrogen bond analysis revealed that **TTh2** exhibited a dynamic yet stabilizing interaction pattern with the *KRAS* G-quadruplex DNA throughout the 200 ns simulation. Initially, the complex maintained 1–2 hydrogen bonds, indicating a transient early interaction phase. However, after approximately 130 ns, a notable increase in hydrogen bonding was observed, with the number of hydrogen bonds stabilizing around 3–4 and occasionally reaching up to 5. This sustained interaction in the latter part of the simulation suggests that **TTh2** gradually adopts a more favorable binding orientation, likely within the grooves of the G4 structure. The persistence and enhancement of hydrogen bonding interactions indicate a key role in anchoring the ligand and maintaining complex stability, reinforcing the hypothesis that **TTh2** is securely accommodated within the DNA groove and contributes to its structural integrity. Collectively, these findings suggest that **TTh2** is a promising G-quadruplex groove-binding ligand capable of stabilizing the *KRAS* G4 motif without disrupting its native topology. This supports its potential utility in targeting G4 structures in cancer research.

Table S3. Bond distances and types of TTh2 with *KRAS* DNA (PDB Id 6T2G) during grove binding.

No.	Molecule Name **TTh2**	Distance (Å)	Bond category	Bond type
1	:dG27:H22 - :**TTh2**:N4	1.84724	Hydrogen Bond	Conventional Hydrogen Bond
2	:**TTh2**:H19 - :dG26:O4'	1.9009	Hydrogen Bond	Conventional Hydrogen Bond
3	:dA1:H8 - :**TTh2**:O3	2.25515	Hydrogen Bond	Carbon Hydrogen Bond
4	:**TTh2**:H21 - :dG26:O1P	2.26347	Hydrogen Bond	Conventional Hydrogen Bond
5	:dG28:H22 - :**TTh2**:O1	2.37748	Hydrogen Bond	Conventional Hydrogen Bond
6	:**TTh2**:H1 - :dT8:O4	2.41247	Hydrogen Bond	Conventional Hydrogen Bond
7	:dG26:H22 - :**TTh2**:N7	2.93786	Hydrogen Bond	Conventional Hydrogen Bond
8	:**TTh2**:HC - :dG28:O1P	2.95337	Hydrogen Bond	Carbon Hydrogen Bond
9	:dG26:H22 - :**TTh2**:N5	3.01018	Hydrogen Bond	Conventional Hydrogen Bond
10	:dG4:H8 - :**TTh2**:O1	3.02676	Hydrogen Bond	Carbon Hydrogen Bond
11	:dG26:H21 - :**TTh2**:N5	3.0676	Hydrogen Bond	Conventional Hydrogen Bond
12	:dG26 - :**TTh2**	3.88366	Hydrophobic	Pi-Pi Stacked
13	:**TTh2**:S2 - :dG26	3.9048	Other	Pi-Sulfur
14	:dG2:O2P - :**TTh2**	4.09364	Electrostatic	Pi-Anion
15	:**TTh2** - :dG26	4.65604	Hydrophobic	Pi-Pi Stacked

12.0 Cell culture and cytotoxicity assay

HeLa cells (human cervical cancer cells) were cultured in DMEM with high glucose (5.5 mM) while HEK cells (human embryonic kidney cells) were incubated in DMEM with 10% FBS at pH 7.4. Both types of cells were cultured in tissue-culture plates at a density of 4×10^5 cells per well at 37 °C in an atmosphere containing 5% CO_2 and 95% air, over a period of 24 hours. To evaluate the in vitro activities of **TTh1** and **TTh2**, various concentration of ligands was applied on cells and incubated another 24 hours before analysis under the same conditions.

The MTT assay is a colorimetric technique used to assess cell viability, measuring the metabolic activity of living cells. This involves the conversion of tetrazolium salt MTT, a yellow and water-soluble compound known as 3-(4,5-dimethylthiazol-2-yl)-2,5-diphenyl tetrazolium bromide, into purple formazan crystals that are insoluble in mitochondria and are basically mitochondrial enzymes of metabolically active cells. This reduction occurs only in viable cells,

giving it an indication of cellular activity. In this assay, HeLa and HEK cell lines were plated in 96-well plates and incubated with different concentrations of **TTh1** and **TTh2** for 24 hours. After 24-hour treatment, media was removed and each well was treated with 100 µL of MTT solution (1 mg/mL in PBS). The plates were then incubated for 4 hours at 37 °C to allow for the development of formazan crystals. Following this, the solution was gently removed, and 100 µL of DMSO was added to each well, dissolving the formazan product. The absorbance of the resulting purple solution was measured at 570 nm using a microplate reader. The percentage of viable cells was calculated based on the absorbance values, indicating the effects of **TTh1** and **TTh2** on the cell viability.

The half maximal inhibitory concentration (IC_{50}) was determined from the graph.

$$Viable\ cells\ (\%) = \frac{A\ of\ treated\ cells}{A\ of\ untreated\ cells}\ X\ 100$$

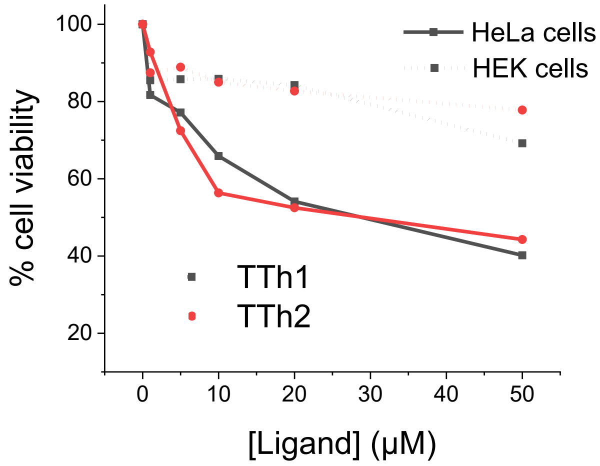

Figure S14. Cytotoxicity assay of **TTh1** and **TTh2** in HeLa cell line (solid line) and HEK cell lines (dotted line).

13.0 RNA extraction and RT-PCR experiments

HeLa cells were treated with less the IC_{50} concentrations of **TTh1** and **TTh2** ligands and incubated for 24 hours. Total RNA was isolated using TRIzol reagent (Invitrogen Corporation). Reverse transcription was performed using the Verso cDNA Synthesis Kit (Thermo Scientific). The master mix for cDNA synthesis consisted of 4 µL of cDNA synthesis buffer, 2 µL of dNTP mix, 1 µL of RNA primer, 1 µL of RT enhancer, 1 µL of Verso enzyme mix, 1 ng from the template RNA and final volume was adjusted to 20 µL with nuclease-free water. Thermal cycling conditions were the following: one cycle of 30 minutes at 42 °C followed by 2 minutes at 95 °C. The cDNA product was then used as a template for quantitative real-time PCR (qRT-PCR) performed on a LightCycler 480 II system (Roche) using SYBR Green JumpStart Taq ReadyMix (Sigma, St. Louis, USA). The PCR reaction mixture (15 µL) consisted of 15 pmol of each primer, 3 µL of cDNA, 7.5 µL of 2X JumpStart Taq ReadyMix, and nuclease-free water, adjusted to a final volume of 15 µL. The primers sequences used for real time RT-PCR are:

KRAS Forward: 5'-$GACGA_2TATGATC_2A_2CA_2TAGAG_2AT_2C$-3'

KRAS Reverse: 5'-$TAG_2TACATCT_2CAGAGTC_2T_2A_2CTC$-3'

18S rRNA Forward: 5'-$GAT_2C_2GTG_4TG_2TG_2TGC$-3'

18S rRNA Reverse: 5'-$A_2GA_2GT_2G_5ACGC_2GA$-3'

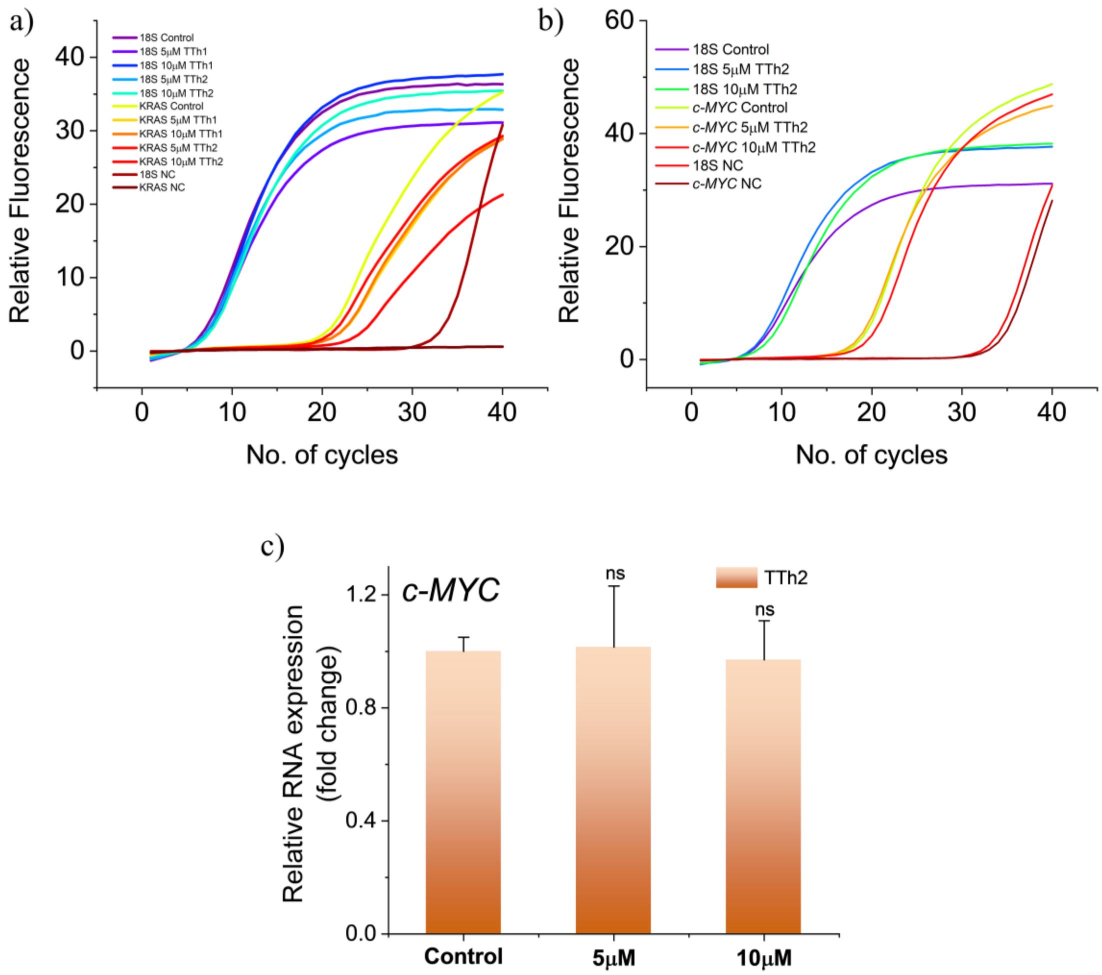

Figure S15.a) qRT-PCR data showing Ct values of *KRAS* gene upon treatment with **TTh1** and **TTh2** in HeLa cells. b) qRT-PCR data showing Ct values of *c-MYC* gene upon treatment with **TTh2** in HeLa cells. c) Bar plot showing fold change in *c-MYC* gene expression in HeLa cells after treatment of 5 and 10μM **TTh2**.

The thermal cycle condition was: pre-incubation at 95 °C for 2 min, followed by 40 cycles of 95 °C for 15 s, 60 °C for 60 s, 72 °C for 90 s, then hold at 37 °C for 2 min.

Then, following equation was used to calculate the ΔCt values:

$$\Delta Ct = Ct\ (target) - Ct\ (reference) \ldots\ldots (i)$$

In our experiment, *KRAS* was the target gene and the 18s rRNA was the reference gene.

Then the ΔΔCT values were calculated by:

$$\Delta\Delta Ct = \Delta Ct\ (test\ sample) - \Delta CT\ (calibrator\ sample) \ldots. (ii)$$

In this experiment, the ΔCt values of the untreated were the calibrator samples and the ΔCT values of the treated were the test sample. Finally, we used the following arithmetic calibrator to calculate the relative level of mRNA expression of the target genes:

$$2^{-(\Delta\Delta Ct)} \ldots\ldots (iii)$$

Three biological replicates were employed for the quantifications. The significance level was statistically analyzed by employing one-way ANOVA test, and results were statistically significant when $^{*}P < 0.001$.

14.0 Western blot

Cells were harvested 24 hours after ligand treatment, washed twice with ice-cold PBS, and lysed on ice for 30 minutes in a cold cell lysis buffer (20 mM Tris, 100 mM NaCl, 1 mM EDTA, and 0.5% Triton X-100). Lysates were cleared of debris by centrifuging at 12,000g for 15 minutes, and the supernatants were pooled for determining total protein on the Folin-Lowry method. Equivalent amounts of protein (50 µg) were resolved on a 12% SDS-PAGE and transferred onto nitrocellulose membranes by electrophoretic transfer. The membranes were blocked with 4% BSA for 2 hours, followed by incubation with specific primary antibodies overnight at 4 °C. Following three washes with 1X TBST, the membranes were exposed for 2 hours to HRP-conjugated secondary antibodies. The blots were washed three times with 1X TBST, once with 1X TBS, and visualized with the HRP substrate. Band intensities were analyzed using the ImageJ software.

Primary Antibodies used:

Anti-KRAS antibody - mouse origin (Santa Cruz)

Anti-GAPDH antibody - rabbit origin (Cell Signaling Technology)

Anti-Akt antibody - rabbit origin (Cell Signaling Technology)

Anti-mTOR antibody - rabbit origin (AB clonal)

Anti-ERK antibody - mouse origin (Santa Cruz)

Anti-p-ERK antibody - rabbit origin (Cell Signaling Technology)

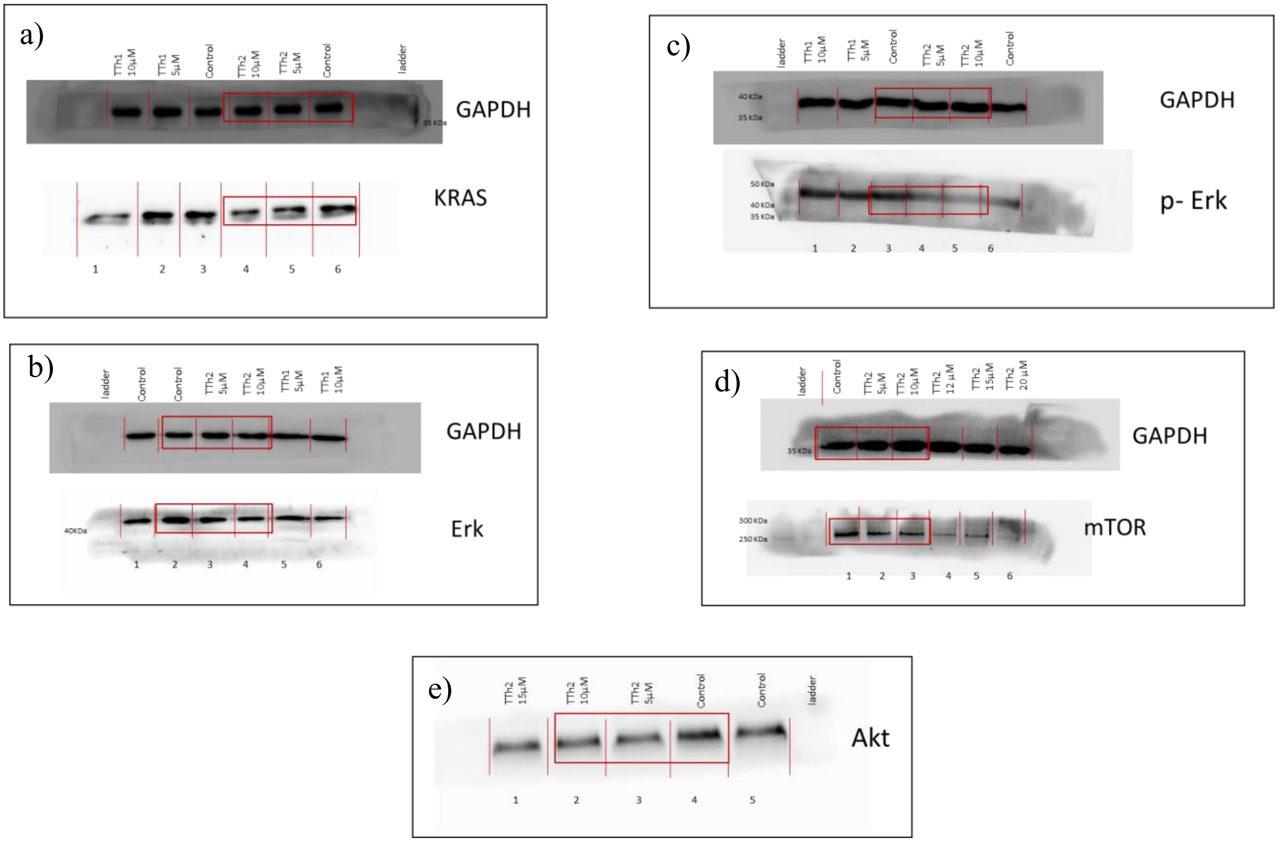

Figure S16. Western blot analysis of a) KRAS, b) Erk, c) phospho-Erk, d) mTOR and e) Akt protein expression in Hela cells treated with **TTh1** or **TTh2** for 24 hours. Lanes 1–6 represent different experimental conditions. The boxed regions indicate the portions displayed in the manuscript. Densitometric analysis of immunoreactive bands was performed, and fold changes

were normalized to the control (untreated) value set at 0. Error bars represent the mean ± SD. *P < 0.05 (Student's t-test) compared to control HeLa cells.

15.0 Transfection and dual luciferase assay

HeLa cells were plated on six-well plates and incubated for 16 hours at 37 °C in a humidified 5% CO_2 atmosphere. Lipofectamine 2000 was used for transfection. Each well received in a 10:1 ratio (PGL3-*KRAS*: pRL-TK) 0.3 µg of PGL3-*KRAS* WT (Addgene plasmid 13919) promoter or c-MYC promoter (Del4) plasmid in combination with 30 ng of pRL-TK (Renilla luciferase). Plasmids were diluted in 0.5 mL DMEM without FBS. Meanwhile, 1 µL of Lipofectamine 2000 (Invitrogen) was diluted in another 0.5 mL of DMEM. Both of these were incubated for 5 minutes at room temperature. The solutions of DNA and Lipofectamine 2000 were combined, in a 1:1 ratio (v/v), mixed by gentle inversion, and incubated at room temperature for 40 minutes forming DNA:Lipofectamine complexes following the manufacturer's protocol. These complexes were then added to approximately 50% confluent cells in the six-well plates. The cells were then incubated at 37 °C in a humidified atmosphere of 5% CO_2 for 6 hours. After the incubation, the media were replaced with 2 mL of DMEM with 10% FBS and 5 and 10µM **TTh1** and **TTh2**. The cells were incubated for an additional 40 hours at 37 °C in a humidified atmosphere of 5% CO_2. Firefly luciferase expression was determined and normalized to Renilla luciferase expression to ensure consistent transfection efficiency.

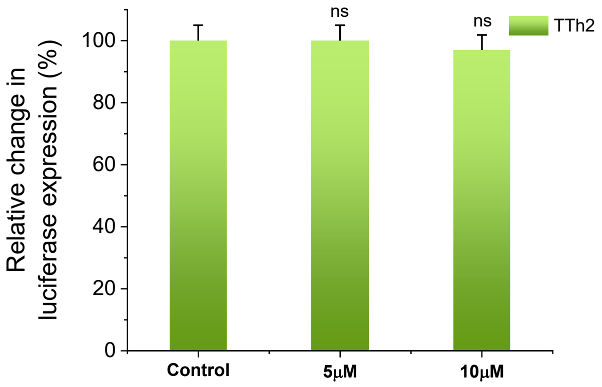

Figure S17. Relative luciferase activity (FF/RL) from the *c-MYC* promoter-driven firefly luciferase plasmid normalized with the pRL-TK Renilla plasmid in HeLa cells treated with 5 µM and 10 µM **TTh2.**

16.0 Immunocytochemistry

Cells cultured on glass coverslips were un-treated as controls and treated with 10 μM of **TTh2** compound for 24 hours. After 24 hours, the cells were washed in 1X PBS, fixed in chilled acetone-methanol (1:1), and permeabilized in 0.03% saponin/PBS. After blocking with 3% BSA in 1X PBS, immuno fluorescence staining was done following standard protocols. The cells were incubated overnight at 37 °C with BG4 antibody (mouse monoclonal, Merck) and *KRAS* antibody (mouse monoclonal, Invitrogen) diluted 1:200 in 1X PBS, followed by a 2-hour incubation during the next day with Alexa Fluor 647-conjugated and Alexa Fluor 488-conjugated secondary antibody (Invitrogen). Coverslips were mounted with Antifade solution (Invitrogen). BG4 fluorescence emission (570-670 nm) was captured using excitation at 559 nm. KRAS fluorescence emission (525 nm) was captured using excitation at 490 nm. Digital images were obtained using a Leica DMI8 Stellaris 5 microscope (scale bars, 10 μm) for BG4 focii visualization and Leica DMI8 microscope (scale bars, 50 μm) for KRAS focii visualization. BG4 and KRAS foci quantifications were made on >50 cells using ImageJ software and average standard error was determined from three different replicates. Statistical significance was determined using Student's t-test and significance was taken as *P < 0.05.

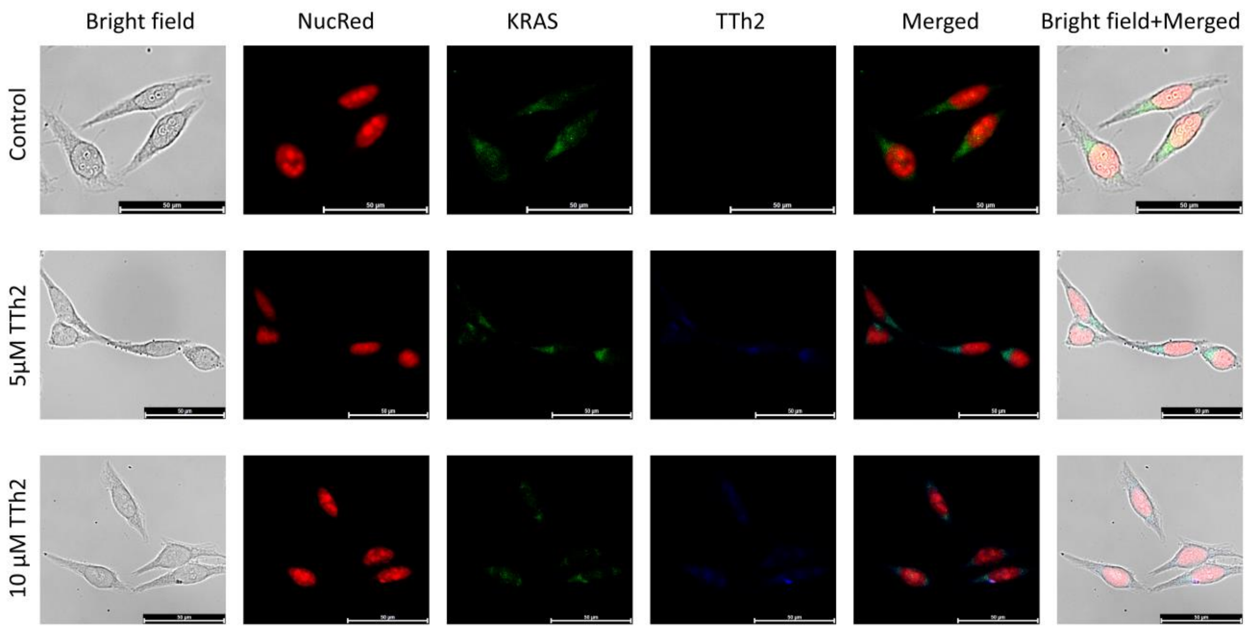

Figure S18. Immunocytochemistry of KRAS protein expression in HeLa cells. The assay shows a dose-dependent decrease in green fluorescence, representing KRAS protein, within the cytoplasm with increasing **TTh2** (blue) concentrations. Nuclei are counterstained with NucRed (red signal) as a reference for nuclear regions, imaged using a Leica DMI8 microscope. Scale bars: 50 μm.

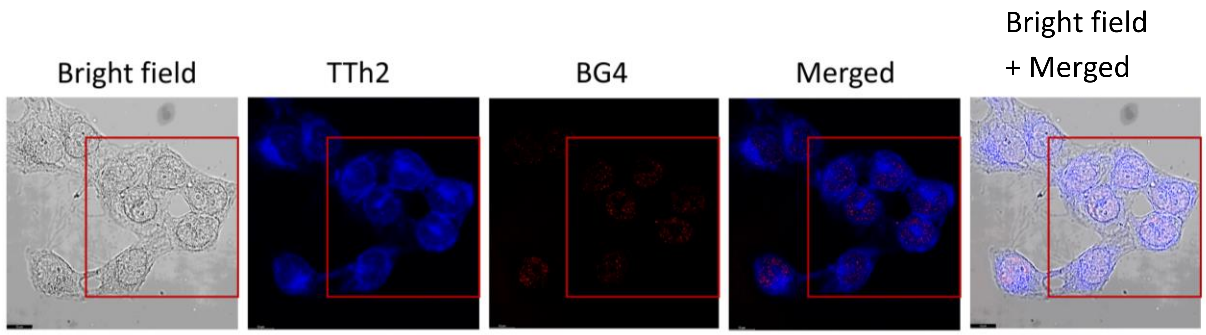

Figure S19. Immunocytochemistry showing nuclear localization of **TTh2** (5μM) and BG4 foci after treatment with **TTh2** in HeLa cells, imaged using a Leica DMI8 Stellaris 5 microscope. Scale bars: 10 μm. The red box indicates the region that has been cropped and presented in the main manuscript figure.

References:

1. R. P. Karuvalam, K. R. Haridas, S. K. Nayak, T. N. G. Row, P. Rajeesh, R. Rishikesan and N. S. Kumari, *European journal of medicinal chemistry*, 2012, **49**, 172-182.

2. K. Nicolaou, D. Rhoades, Y. Wang, R. Bai, E. Hamel, M. Aujay, J. Sandoval and J. Gavrilyuk, *Journal of the American Chemical Society*, 2017, **139**, 7318-7334.

3. D. Dutta, M. Debnath, D. Müller, R. Paul, T. Das, I. Bessi, H. Schwalbe and J. Dash, *Nucleic acids research*, 2018, **46**, 5355-5365.

4. G. Chakraborti, S. Paladhi, T. Mandal and J. Dash, *The Journal of Organic Chemistry*, 2018, **83**, 7347-7359.

5. Kolpakov FA, Babenko VN. Computer system MGL: tool for sample generation, visualization and analysis of regulatory genomic sequences. Molecular Biology. 1997 Jul 1;31(4):540-7.

6. Trott O, Olson AJ. AutoDock Vina: improving the speed and accuracy of docking with a new scoring function, efficient optimization, and multithreading. Journal of computational chemistry. 2010 Jan 30;31(2):455-61.

7. Brooks III CL, Karplus M. Solvent effects on protein motion and protein effects on solvent motion: dynamics of the active site region of lysozyme. Journal of molecular biology. 1989 Jul 5;208(1):159-81.

8. Zoete V, Cuendet MA, Grosdidier A, Michielin O. SwissParam: a fast force field generation tool for small organic molecules. Journal of computational chemistry. 2011 Aug;32(11):2359-68.

9. Darden T, York D, Pedersen L. Particle mesh Ewald: An N· log (N) method for Ewald sums in large systems. The Journal of chemical physics. 1993 Jun 15;98(12):10089-92.

10. Grant, B. J., Rodrigues, A. P. C., ElSawy, K. M., McCammon, J. A., & Caves, L. S. D. (2006). Bio3D: an R package for the comparative analysis of protein structures. Bioinformatics, 22(21), 2695–2696.

11. Leach, A. R. (2001). Molecular Modelling: Principles and Applications (2nd ed.). Pearson Education.

12. Amadei, A., Linssen, A. B. M., & Berendsen, H. J. C. (1993). Essential dynamics of proteins. Proteins: Structure, Function, and Bioinformatics, 17(4), 412–425.

13. Hollingsworth, S. A., & Dror, R. O. (2018). Molecular dynamics simulation for all. Neuron, 99(6), 1129–1143.

14. Fleming, P. J., & Fleming, K. G. (2018). HullRad: Fast Calculations of Folded and Disordered Protein and Nucleic Acid Hydrodynamic Properties. Biophysical Journal, 114(4), 856–869.

15. Schlick, T. (2010). Molecular Modeling and Simulation: An Interdisciplinary Guide (2nd ed.). Springer. ISBN: 978-1-4419-6350-6.
